# Empirical Bayesian LASSO-logistic regression for multiple binary trait locus mapping

**DOI:** 10.1186/1471-2156-14-5

**Published:** 2013-02-15

**Authors:** Anhui Huang, Shizhong Xu, Xiaodong Cai

**Affiliations:** 1 Department of Electrical and Computer Engineering, University of Miami, Coral Gables, FL 33146, USA; 2 Department of Botany and Plant Sciences, University of California, Riverside, CA 92521, USA

**Keywords:** QTL mapping, Binary traits, Epistatic effects, Bayesian shrinkage, Logistic regression

## Abstract

**Background:**

Complex binary traits are influenced by many factors including the main effects of many quantitative trait loci (QTLs), the epistatic effects involving more than one QTLs, environmental effects and the effects of gene-environment interactions. Although a number of QTL mapping methods for binary traits have been developed, there still lacks an efficient and powerful method that can handle both main and epistatic effects of a relatively large number of possible QTLs.

**Results:**

In this paper, we use a Bayesian logistic regression model as the QTL model for binary traits that includes both main and epistatic effects. Our logistic regression model employs hierarchical priors for regression coefficients similar to the ones used in the Bayesian LASSO linear model for multiple QTL mapping for continuous traits. We develop efficient empirical Bayesian algorithms to infer the logistic regression model. Our simulation study shows that our algorithms can easily handle a QTL model with a large number of main and epistatic effects on a personal computer, and outperform five other methods examined including the LASSO, HyperLasso, BhGLM, RVM and the single-QTL mapping method based on logistic regression in terms of power of detection and false positive rate. The utility of our algorithms is also demonstrated through analysis of a real data set. A software package implementing the empirical Bayesian algorithms in this paper is freely available upon request.

**Conclusions:**

The EBLASSO logistic regression method can handle a large number of effects possibly including the main and epistatic QTL effects, environmental effects and the effects of gene-environment interactions. It will be a very useful tool for multiple QTLs mapping for complex binary traits.

## Background

Quantitative traits are usually influenced by multiple quantitative trait loci (QTLs), environmental factors and their interactions [1]. Although complex binary traits only show binary phenotypic variation different from the continuous variation in quantitative traits, they do not follow a simple Mendelian pattern of inheritance and also have a polygenic basis similar to that of quantitative traits. Therefore, like QTL mapping for quantitative traits, mapping for complex binary traits aims to identify multiple genomic loci that are associated with the trait and to estimate the genetic effects of these loci possibly including any of the following effects: main effects, gene-gene interactions (epistatic effects) and effects of gene-environment interactions.

A number of statistical methods have been developed to identify QTLs for binary traits in experimental crosses. Single-QTL mapping methods [2-8] analyze the association between each individual genetic locus and the trait independently. However, single-QTL mapping method can only find the main effect of a QTL and cannot detect epistatic effects involving more than one locus. Moreover, it has been shown both theoretically and empirically that multiple-QTL methods can improve power in detecting QTLs and eliminate possible biases in the estimates of QTL locations and genetic effects introduced by a single-QTL model [9,10]. Therefore, several multiple QTL mapping for binary traits have been developed. These include Bayesian methods [11-16] that rely on Markov Chain Monte Carlo (MCMC) simulation to infer a multiple QTL threshold model for binary, ordinal or longitudinal traits, the multiple-interval mapping (MIM) methods [17,18] that use the expectation-maximization (EM) algorithm to infer the threshold model or a generalized linear model for binary or ordinal traits, and a method [19] that employs a probability model based on classical transmission genetics to identify binary trait loci. However, all these methods require large computation when the QTL model includes a relatively large number of loci.

Since hundreds or thousands of genomic loci or markers are usually genotyped and involved in QTL mapping studies, including all these markers and their possible interactions in a single model for multiple-QTL mapping leads to a huge number of model variables, typically much larger than the sample size. This not only entails huge computation that is not affordable to existing QTL mapping methods mentioned earlier but also may reduce power of detection and/or increase false discover rate. Two techniques have been proposed to handle the second problem: variable or model section and shrinkage. More specifically, model selection using the Akaike or Bayesian information criterion (AIC or BIC) and variable selection based on stepwise logistic regression and the BIC have been proposed in [19] and [18], respectively, to restrict the model space and to reduce the number of variables included in the model. Bayesian shrinkage method that was first applied to QTL mapping for continuous traits [15,20-24] has also been used in QTL mapping for binary traits [15,22], which employed MCMC for the inference of the QTL model. To reduce computational burden, more efficient methods [25,26] were developed to infer Bayesian QTL models for binary traits. Another well-known method for shrinking variables is the least absolute shrinkage and selection operator (LASSO) [27], which has been applied to QTL mapping for continuous traits [24] and investigated for genome-wide association study (GWAS) of complex diseases [28,29].

Recently, we developed an efficient empirical Bayesian LASSO (EBLASSO) algorithm for multiple-QTL mapping for continuous traits, which is capable of handling a large number of markers and their interactions simultaneously [30]. In this paper, we extend the linear Bayesian LASSO model [23,30,31] to logistic regression to map multiple QTLs for binary traits. We consider a three-level and a two-level hierarchical model for the prior distributions of the regression coefficients. Building on the EBLASSO algorithm [30], we develop efficient empirical Bayesian algorithms to infer the Bayesian LASSO logistic regression model with two different priors. We then use simulations to compare the performance of our EBLASSO with that of five other QTL mapping methods for binary traits, that include the LASSO-logistic regression [27,28], the HyperLasso [25], the Bayesian hierarchical generalized linear models (BhGLM) [26], the relevant vector machine (RVM) [32,33], and the single-QTL mapping method based on logistic regression. Simulation results show that our EBLASSO offers best overall performance among the examined methods in terms of power of detection and false positive rate. Analysis of a real dataset with our algorithms also identifies several QTLs.

## Methods

### Logistic regression model

Let *y*_*i*_ = 0 or 1 denote the binary trait of the *i*th sample of *n* individuals in a study. Let us define ***y*** = [*y*_*1*_*, y*_*2*_*,* ···*, y*_*n*_]^*T*^ as the binary phenotypes for all the *n* individuals. The probability of observing *y*_*i*_ = 1 is written as *p*_*i*_ = Pr(*y*_*i*_ = 1), *i* = 1, ···, *n*, which are further collected into a vector ***p*** = [*p*_*1*_*, p*_*2*_*,* ···*, p*_*n*_]^*T*^. Suppose that *m* genetic markers of these *n* individuals are genotyped and the genotype of marker *j* of individual *i* is *x*_*ij*_. Taking main and epistatic effects of all markers into consideration, we have the following logistic regression model for multiple-QTL mapping:

(1) $$\mathrm{logit}\left(p_{i}\right)=\beta_{0}+\sum_{j=1}^{m}\beta_{j}x_{\mathrm{ij}}+\sum_{j=1}^{m-1}\sum_{j^{\prime }>j}^{m}\beta_{jj^{\prime }}x_{\mathrm{ij}}\cdot x_{ij^{\prime }}$$

where *β*_*j*_ and *β*_*jj’*_ are regression coefficients, and logit(*p*_*i*_) is defined as:

(2) $$\mathrm{logit}\left(p_{i}\right)=\log\left[\frac{\mathrm{Pr}\left(y_{i}=1\right)}{1-\mathrm{Pr}\left(y_{i}=1\right)}\right]$$

The widely adopted Cockerham genetic model [34] will be used in this paper. For a back-cross design, the Cockerham model assigns −0.5 and 0.5 to *x*_*ij*_ for two possible genotypes at marker *j*. For an intercross (F_2_) design, there are two possible main effects named additive and dominance effects. The Cockerham model defines the values of the additive effect as −1, 0 and 1 for the three genotypes and the values of the dominance effect as −0.5 and 0.5 for homozygotes and heterozygotes, respectively. For simplicity, we only consider additive effects in (1), although the methods developed in this paper are also applicable to the model with dominance effects.

Let us define **x**_*Gi*_ = [*x*_*i*1_, *x*_*i*2_, ⋯, *x*_*im*_]^*T*^, and $\beta_{G}={\left[\beta_{1},\beta_{2},\cdot \cdot \cdot ,\beta_{m}\right]}^{T}$. Let **x**_*GGi*_ be a *m*(*m* − 1)/2 × 1 vector containing $x_{\mathrm{ij}}\cdot x_{ij^{\prime }},j=1,\cdots ,m-1,j^{\prime }>j$, and ***β***_*GG*_ be a vector consisting of corresponding regression coefficients. We further define **x**_*i*_ = [1, **x**_*Gi*_^*T*^, **x**_*GGi*_^*T*^]^*T*^ and $\beta ={\left[\beta_{0},\beta_{G}^{T},\beta_{\mathrm{GG}}^{T}\right]}^{T}$, then (1) can be written in a more compact form:

(3) $$\mathrm{logit}\left(p_{i}\right)=x_{i}^{T}\beta$$

From (3), we can express *p*_*i*_ as follows:

(4) $$p_{i}=\frac{1}{1+e^{-x_{i}^{T}\beta }}$$

Note that there are *k* = 1 + *m*(*m* + 1)/2 unknown regression coefficients in (1) or (3). Typically, we have *k* ≫ *n*. If dominance effects of the markers are considered, *k* is even larger. Simultaneously estimation of all possible genetic effects with *k* ≫ *n* is a challenging problem. However, we would expect that most elements of ***β*** are zeros and thus we have a sparse model. We will exploit this sparsity to develop efficient methods to infer ***β***.

#### Prior distributions for the regression coefficients

We assign a noninformative uniform prior to *β*_0_, i.e., *p*(*β*_0_) ∝ 1. For *β*_*i*_, *i* = 1, 2,···, *k*, we will consider two hierarchical models for the prior distribution. The first model is the one used in both the linear Bayesian LASSO model [23,30,31] and the HyperLasso logistic regression [25,35], which has three levels. At the first level, *β*_*i*_, *i* = 1, 2,···, *k*, follows an independent normal distribution with mean zero and variance *σ*_*i*_^2^: *β*_*i*_ ∼ *N*(0, *σ*_*i*_^2^). At the second level, *σ*_*i*_^2^, *i* = 1, 2, ···, *k*, follows an independent exponential distribution with probability density function *p*(*σ*_*i*_^2^) = *λ*exp(−*λσ*_*i*_^2^), with parameter *λ* > 0. At the third level, *λ* follows a gamma distribution *gamma*(*a*, *b*) with a shape parameter *a* and an inverse scale parameter *b*. We name the logistic regression model with this normal-exponential-gamma (NEG) prior as BLASSO-NEG.

The three-level hierarchical model has two hyperparameters *a* and *b*. While these two parameters give much flexibility of adjusting the degree of shrinkage, significant computation is required for cross validation to properly choose their values. To reduce the computational burden of cross validation, we will also consider a model with only the first two levels in the BLASSO-NEG. This two level hierarchical model only has one hyperparameter *λ* to be adjusted, and thus, it requires less computation. We name the logistic regression model with the two-level prior as BLASSO-NE. We will next develop two empirical Bayes (EB) algorithms to infer these two models.

### Empirical Bayesian algorithm for the BLASSO-NEG model (EBLASSO-NEG)

Using the EB approach [36], we will first estimate *σ*_*i*_^2^, *i* = 1, 2,⋯, *k*, from the data, and then find the posterior distribution of ***β*** based on the estimated *σ*_*i*_^2^. As shown in [30], the prior distribution of *σ*_*i*_^2^ can be found as

(5) $$p\left(\sigma_{i}^{2}\right)=\int_{0}^{\infty }p\left(\sigma_{i}^{2},|,\lambda \right)p\left(\lambda \right)\mathrm{d\lambda}=\frac{a}{b{\left(\sigma_{i}^{2}/b+1\right)}^{a+1}}$$

Let us define $\sigma^{2}={\left[\sigma_{1}^{2},\sigma_{2}^{2},\cdot \cdot \cdot ,\sigma_{k}^{2}\right]}^{T}$ and ***y*** = *y*_*1*_*, y*_*2*_*,* ···*, y*_*n*_^*T*^, then the posterior distribution of ***β*** and ***σ***^*2*^ is given by:

(6) $$p\left(\beta ,\sigma^{2}|y\right)\propto p\left(y|\beta \right)p\left(\beta |\sigma^{2}\right)p\left(\sigma^{2}\right)$$

where $p\left(y|\beta \right)=\prod_{i=1}^{n}p_{i}^{y_{i}}{\left(1-p_{i}\right)}^{1-y_{i}}$ and $p\left(\beta |\sigma^{2}\right)$ is a normal distribution. Since it is difficult to integrate out ***β*** in (6) to get the marginal posterior distribution of ***σ***^*2*^, it is difficult to estimate ***σ***^*2*^ directly by maximizing its posterior function. To overcome this problem, we will employ an iterative approach that relies on Laplace approximation of the posterior distribution of ***β***[32,37,38].

Let *α*_*i*_ = 1/*σ*_*i*_^2^, *i* = 1, 2,⋯, *k*, and collect them in a vector $\alpha ={\left[\alpha_{1},\alpha_{2},\cdot \cdot \cdot ,\alpha_{k}\right]}^{T}$. Then, we have $\beta ∼N\left(0,A^{-1}\right)$, where **A** is a diagonal matrix With ***α*** on its diagonal. Suppose in the (*i*-1)th iteration, we have estimated **A** as **Â**. Given ***y*** and **A** = **Â**, the posterior distribution of ***β*** can be approximated by a Gaussian distribution found with the Laplace approximation approach [37] as follows. Since the posterior distribution of ***β*** is given by $p\left(\beta |y\right)\propto p\left(y|\beta \right)p\left(\beta \right)$, we have:

(7) $$\begin{array}{l} \log p\left(\beta |y\right)=\sum_{i=1}^{n}\left[y_{i}\log p_{i}+\left(1-y_{i}\right)\log\left(1-p_{i}\right)\right]\\ \;-\frac{1}{2}\beta^{T}\text{A}\beta +\mathrm{constant} \end{array}$$

The gradient ***g*** and Hessian matrix **H** of $\log p\left(\beta |y\right)$ are given by $g=X^{T}\left(y-p\right)-A\beta$ and **H** = − (***X***^*T*^**BX** + **A**), respectively, where ***p*** = *p*_*1*_*, p*_*2*_*,*⋯*, p*_*n*_^*T*^, **X** = **x**_1_^*T*^, **x**_2_^*T*^, ⋯, **x**_*n*_^*T*^^*T*^ and **B** is a diagonal matrix with the diagonal entries *p*_1_(1 − *p*_1_), ⋅ ⋅⋅, *p*_*n*_(1 − *p*_*n*_). With ***g*** and **H** available, we can use the Newton–Raphson method to find the maximum *a posterior* (MAP) estimate or the mode of ***β***, by maximizing $\log p\left(\beta |y\right)$, which is denoted as ${\hat{\beta }}_{\mathrm{MAP}}$. Then the Laplace approximation of $p\left(\beta |y,\hat{\text{A}}\right)$ is a normal distribution $\hat{p}\left(\beta |y,\hat{\text{A}}\right)$, with mean ${\hat{\beta }}_{\mathrm{MAP}}$ and covariance given by [37]:

(8) $$\Sigma ={\left(-H\right)}^{-1}={\left(X^{T}B_{\mathrm{MAP}}X+\hat{\text{A}}\right)}^{-1}$$

where **B**_*MAP*_ is obtained with ${\hat{\beta }}_{\mathrm{MAP}}$.

If we postulate a linear model ${\hat{y}}^{=X\beta }+\varepsilon$ε, where $\hat{y}=X{\hat{\beta }}_{\mathrm{MAP}}+B_{\mathrm{MAP}}^{-1}\left(y-p_{\mathrm{MAP}}\right)$ with ***p***_*MAP*_ being obtained with ${\hat{\beta }}_{\mathrm{MAP}}$, $\varepsilon ∼N\left(0,B_{\mathrm{MAP}}^{-1}\right)$ and $\beta ∼N\left(0,{\hat{\text{A}}}^{-1}\right)$, we can show that $\hat{p}\left(\beta |y,\hat{\text{A}}\right)$ is the posterior distribution of ***β*** in this linear model as follows. The linear model implies that the posterior distribution of ***β*** is normal with mean $\Sigma X^{T}B_{\mathrm{MAP}}\hat{y}$ and covariance **Σ**[37]. Hence, we need to prove ${\hat{\beta }}_{\mathrm{MAP}}=\Sigma X^{T}B_{\mathrm{MAP}}\hat{y}$. Since the gradient ***g*=** 0 at ***β***_*MAP*_, we have $\hat{\text{A}}{\hat{\beta }}_{\mathrm{MAP}}=X^{T}\left(y-p_{\mathrm{MAP}}\right).$From (8), we get $\hat{\text{A}}=\Sigma^{-1}-X^{T}B_{\mathrm{MAP}}X,$Therefore, we have $\left(\Sigma^{-1}-X^{T}B_{\mathrm{MAP}}X\right){\hat{\beta }}_{\mathrm{MAP}}=X^{T}\left(y-p_{\mathrm{MAP}}\right)$, which implies that $\Sigma^{-1}{\hat{\beta }}_{\mathrm{MAP}}=X^{T}B_{\mathrm{MAP}}\left[X{\hat{\beta }}_{\mathrm{MAP}}-B_{\mathrm{MAP}}^{-1}\left(y-p_{\mathrm{MAP}}\right)\right]=X^{T}B_{\mathrm{MAP}}\hat{y}$. This leads to ${\hat{\beta }}_{\mathrm{MAP}}=\Sigma X^{T}B_{\mathrm{MAP}}\hat{y}$ as desired.

Therefore, in the *i*th iteration we form the following linear model:

(9) $$\hat{y}=X\beta +\varepsilon$$

where ***ŷ*** and the distribution of ***ε*** are defined earlier, but ***β*** follows the three-level BLASSO-NEG model. Then based on the linear model (9), we use the EBLASSO algorithm [30] to get a new estimate of **A**, which replaces **Â** obtained in the (*i*-1)th iteration. The iteration process goes on until certain convergence criterion is satisfied, which gives the final estimate of **A** and Laplace approximation $\hat{p}\left(\beta |y,\hat{A}\right)$ of the posterior distribution of ***β***. The iterative process needs to be initialized. Similar to the EBLASSO [30], we assume that only one regression coefficient is initially nonzero or equivalently only one *α*_*i*_ is finite. The EBLASSO-NEG algorithm is summarized as follows:

#### Algorithm 1 (EBLASSO-NEG)

1. Initialization: choose *a* > −1.5 and *b* > 0. The first basis in the model, ***x***_*j*_, is identified by $j=\arg_{i}\max\left\{|x_{i}^{T}\left(y-p_{0}\right)|,\forall i\right\}$, with *p*_*0*_ being the proportion of *y*_*i*_ = 1 in the dataset. Compute ${\hat{\beta }}_{j}={\left({\tilde{x}}_{j}^{T}{\tilde{x}}_{j}\right)}^{-1}{\tilde{x}}_{j}^{T}\left(y-p_{0}\right)$ with ${\tilde{x}}_{j}=x_{j}-\frac{1}{n}\sum_{i=1}^{n}x_{\mathrm{ij}}$, and set $\alpha_{j}=1/{\hat{\beta }}_{j}^{2}$ and all *α*_*i*_, *i ≠ j* notionally to infinity

2. While the convergence criteria are not satisfied

3. Given **Â**, use the Newton–Raphson method to find ${\hat{\beta }}_{\mathrm{MAP}}$

4. Calculate $\hat{y}=X{\hat{\beta }}_{\mathrm{MAP}}+B^{-1}\left(y-p\right)$

5. Apply the EBLASSO algorithm [30] to linear model (9) to update **Â**.

6. End while

7. Output ${\hat{\beta }}_{\mathrm{MAP}}$ and covariance **Σ**.

Note that the algorithm starts with a logistic regression model with only one variable and then iteratively adds variables with a finite *α*_*i*_ to the model. The number of variables in the model *k*_*m*_ is typically much smaller than the total number of possible variables *k*. Since we only need to calculate the gradient ***g*** and the Hessian matrix **H** for the *k*_*m*_ variables in the model, the computation required in step 3 and in the calculation of **Σ** in (8) is relatively small. Moreover, the EBLASSO algorithm in step 5 is very efficient due to the fact that the variance components can be estimated in a closed form and other algorithmic techniques as discussed in [30].

The convergence criteria in Algorithm 1 are defined as: 1) no effect can be added to or delete from the model, 2) the likelihood change between two consecutive iterations is less than a pre-specified small value and 3) the total change of ***α*** between two consecutive iterations is less than a pre-specified small value. In step 5, the EBLASSO algorithm [30] needs to be modified slightly. First, since the noise covariance is known as **B**^-1^, we do not estimate noise covariance. Second, since the mean of ***ŷ*** in (9) is zero, we do not need to estimate it. Third, we use the formula **Σ** = (**X**^*T*^**BX** + **A**)^− 1^ to update the covariance of ***β***.

The values of hyperparameters (*a*, *b*) are determined by cross validation as will be described in the Result section. The first basis in the initialization step is determined using the method in LASSO-logistic regression [28]. The initial value ${\hat{\beta }}_{j}$ of the first basis is calculated from a linear regression model that uses ***y*** as the response variable [39], and the variance *σ*_*j*_^*2*^ is approximated as ${\hat{\beta }}_{j}^{2}$, resulting in $\alpha_{j}=1/{\hat{\beta }}_{j}^{2}$.

Suppose that ${\hat{\beta }}_{\mathrm{MAP}}$ output from the EBLASSO-NEG algorithm contains *k*_*n*_ entries. Then the posterior distribution of the corresponding *k*_*n*_ × 1 regression coefficient $\hat{\beta }$ can be approximated by a normal distribution with mean ${\hat{\beta }}_{\mathrm{MAP}}$ and covariance **Σ**. For the *i*th entry of $\hat{\beta }$, we can calculate a *t*-statistics $t_{i}={\hat{\beta }}_{i}/\Sigma_{\mathrm{ii}}^{1/2}$, and then use the student’s distribution to calculate a *p*-value for ${\hat{\beta }}_{i}$. Markers that correspond to those ${\hat{\beta }}_{i}$ with a *p*-value less than a threshold, say 0.05, are then identified as QTLs and the corresponding entries of ${\hat{\beta }}_{\mathrm{MAP}}$ are estimated effect sizes of the QTLs.

We next derive an efficient EB algorithm for the BLASSO-NE model, which simplifies the hyperparameter selection, since we only need to determine the optimal value of one hyperparameter *λ* using cross validation.

### Empirical Bayesian algorithm for the BLASSO-NE model (EBLASSO-NE)

The prior distribution of *σ*_*i*_^2^, *i* = 1, 2, …, *k* or equivalently *α*_*i*_, *i* = 1, 2, …, *k* is used only in step 5 of the EBLASSO-NEG algorithm. Because the EBLASSO-NE model uses a different prior distribution from the one used in the BLASSO-NEG model, we will derive a new formula for estimating **A** in each iteration. Then the EBLASSO-NE algorithm uses the same steps in the EBLASSO-NEG except that the new formula is used in step 5 to find **Â**.

Suppose that the postulated linear model after step 4 is $\hat{y}=X\beta_{\mathrm{MAP}}+\varepsilon$. Following the derivation in [30], the log marginal posterior distribution of ***α*** can be found as:

(10) $$\begin{array}{c} L\left(\alpha \right)=-\frac{1}{2}\left[\log\left|C\right|+{\hat{y}}^{T}C^{-1}\hat{y}\right]\\ \;-\sum_{i}^{k}\log\frac{\lambda }{\alpha_{i}}+\mathrm{constant} \end{array}$$

where $C\;=XA^{-1}X^{T}+B^{-1}$ is the covariance matrix of **ŷ** in the linear model. Each *α*_*i*_ will be estimated iteratively by maximizing the log marginal posterior distribution *L*(***α***) with the other parameters fixed. Specifically, *L*(***α***) can be written as $L\left(\alpha \right)=L\left(\alpha_{-i}\right)+L\left(\alpha_{i}\right)$, where *L*(***α***) is a function of *α*_*i*_ and $L\left(\alpha_{-i}\right)$ is a function of the remaining parameters. By defining $C_{-i}=C-\alpha_{i}{}^{-1}x_{i}x_{i}^{T}$, we can write *L*(***α***) as:

(11) $$L\left(\alpha_{i}\right)=\frac{1}{2}\left[\log\frac{\alpha_{i}}{\alpha_{i}+s_{i}}+\frac{q_{i}^{2}}{\alpha_{i}+s_{i}}\right]-\frac{\lambda }{\alpha_{i}}$$

where $s_{i}=x_{i}^{T}C_{-i}^{-1}x_{i}$ and $q_{i}=x_{i}^{T}C_{-i}^{-1}\hat{y}$. In the Additional file 1, we show that *L*(***α***) has a unique global maximum and that the optimal *α*_*i*_ maximizing L(***α***) is given by:

(12) $$\alpha_{i}^{*}=\{\begin{array}{c} r,\mathrm{if}\;q_{i}^{2}-s_{i}>2\lambda \\ \infty ,\mathrm{otherwise},\; \end{array}$$

where $r=\frac{-\left(s_{i}+4\lambda \right)-\sqrt{\Delta }}{2\left(s_{i}-q_{i}^{2}+2\lambda \right)}\cdot s_{i}$, and *Δ* = *s*_*i*_^2^ + 8*λq*_*i*_^2^.

The EBLASSO-NE algorithm has the same steps as those in Algorithm 1 but with the following two modifications. First, we need to choose a value for parameter *λ* instead of *a* and *b* in step 1, which can be done using cross validation. In the LASSO-logistic regression [28], an upper bound of *λ* was estimated to be $\lambda_{\mathrm{lasso}}=\arg_{j}\max\left|x_{j}^{T}\left(y-p_{0}\right)\right|$. In our EBLASSO-NE, we suggest the maximum value of *λ* to be *λ*_max_= 1.5*λ*_*lasso*_ based on our simulations showing that this maximum value usually only gives one nonzero regression coefficient. Second, when applying the EBLASSO algorithm in step 5 of Algorithm 1, the EBLASSO algorithm uses equation (12) instead of equation (8) in [30] to estimate **A**.

Note that since the EBLASSO-NEG model for logistic regression considered in this paper uses the same prior as the one used by the EBLASSO for linear regression considered in [30], the EBLASSO-NEG algorithm is essentially a combination of Laplace approximation and the EBLASSO algorithm [30]. The EBLASSO-NE model considered here however uses a prior different from the one used by the EBLASSO [30]. Therefore, we employ (12) to modify the EBLASSO algorithm in [30], and then the EBLASSO-NE algorithm is a combination of Laplace approximation and the modified EBLASSO algorithm.

## Results

### Simulation study

A total of *m* = 481 genetic markers were simulated to be evenly spaced on a large chromosome of 2400 centi-Morgan (cM) with an interval of *d* = 5 cM, which gives rise to a correlation of *R* = e^-2*d*^ = 0.9048 since the Haldane map function [40] was assumed. The simulated population was an F_2_ family derived from cross of two inbred lines. The dummy variable for the three genotypes, *A*_*1*_*A*_*1*_*, A*_*1*_*A*_*2*_ and *A*_*2*_*A*_*2*_ of individual *i* at marker *j* was defined as *x*_*ij*_ = 1, 0, -1, respectively. Two simulation setups were employed. In the first setup, 20 markers were QTLs with main effects but without interactions. In the second setup, 10 main and 10 epistatic effects were simulated; a marker could have both main and epistatic effects, while two markers involving in an interaction effect did not necessarily have main effects. The QTLs were selected randomly with varying distances (5 cM - 500 cM) and effect sizes (in the range between −1.28 and 2.19). Note that QTLs were assumed to be coincided with markers in both simulation setups. If QTLs are not on markers, they may still be detected since correlation between a QTL and a nearby marker is high, although a slightly larger sample size may be needed to give the same power of detection.

EBLASSO-NEG and EBLASSO-NE algorithms were implemented in C and could be called from the R environment [41], and thus QTL mapping with these two algorithms were carried out in R. To compare the performance of our algorithms with that of other relevant algorithms, we also analyzed the simulated data with the following five QTL mapping methods: the LASSO regularized logistic regression implemented in the *glmnet* package [42], the HyperLasso [25], the BhGLM method [26], the RVM [32,33], and the single QTL logistic regression test using the *glm* procedure in R. Simulation results from these methods are presented next.

#### Simulation result for the dataset with only main effects

The genotypes of *m* = 481 markers of *n* = 500 individuals were generated using the procedure described earlier. Twenty markers were chosen as QTLs; their IDs and effect sizes are given in Table 1. Let **x**_*i*_ be a 20 × 1 vector containing the genotypes of 20 QTLs of individual *i*, and ***β*** contain corresponding effect sizes. Then probability *p*_*i*_ was calculated from $p_{i}=1/\left(1+e^{-x_{i}^{T}\beta }\right)$, and *y*_*i*_ was generated from a Bernoulli random variable with parameter *p*_*i*_. Therefore, a simulated data set included a 500 × 1 vector ***y*** and a 500 × 481 design matrix **X**.

**Table 1:** True and estimated effects for the simulated data with main effects

**locus**	**True *β***	**EBLASSO-NE**$\hat{\beta }{\left(s_{\hat{\beta }}\right)}^{a}$	**EBLASSO-NEG**$\hat{\beta }{\left(s_{\hat{\beta }}\right)}^{a}$	**LASSO**$\hat{\beta }{\left(s_{\hat{\beta }}\right)}^{a}$	**HyperLasso**$\hat{\beta }{\left(s_{\hat{\beta }}\right)}^{a}$	**BhGLM**$\hat{\beta }{\left(s_{\hat{\beta }}\right)}^{a}$	**RVM**$\hat{\beta }{\left(s_{\hat{\beta }}\right)}^{a}$	**Single QTL**$\hat{\beta }{\left(s_{\hat{\beta }}\right)}^{a}$
11	1.99	0.76(0.22)	1.61(0.22)	0.51(0.73)	1.67(0.30)	1.50(0.27)	3.87(0.60)	0.67(0.13)
26	1.81	0.54(0.19)	1.23(0.21)	−	0.93(0.38)	1.07(0.28)	1.53(0.47)^*b*^	0.73(0.13)
42	−1.28	−0.34(0.17)	−0.72(0.21)^*b*^	−	−1.02(0.29)	−0.95(0.24)	−	−
48	−0.91	−0.40(0.19)	−0.82(0.21)	−	−1.12(0.28)^*b*^	−0.91(0.23)	−	−
72	1.28	−	−	−	−	−	−	0.77(0.14)
73	1.81	1.37(0.21)	1.91(0.24)	1.03(0.85)	2.37(0.32)	2.16(0.28)	4.73(0.59)	0.80(0.14)
123	0.63	−	−	−	−	−	−	−
127	−0.63	−	−	−	−	−	−	−
161	0.44	0.30(0.15)^*b*^	0.59(0.19)^*b*^	−	0.82(0.25)^*b*^	−	1.15(0.35)	0.57(0.13)
181	0.99	0.38(0.20)^*b*^	−	−	−	−	−	1.67(0.18)
182	2.19	1.60(0.29)	2.86(0.31)	1.26(0.86)	3.34(0.38)	−2.73(0.37)	5.01(0.72)	1.89(0.19)
185	1.29	0.36(0.17)^*b*^	0.56(0.19)^*b*^	0.27(0.43)^*b*^	1.00(0.27)^*b*^	0.73(0.32)	2.38(0.69)^*b*^	1.44(0.16)
221	−0.75	−	−0.36(0.16)^*b*^	−	−	−	−	−
243	−0.57	−0.34(0.15)	−0.41(0.16)	−0.26(0.33)	−0.75(0.24)	−0.69(0.20)	−1.74(0.45)	−
262	−1.28	−	−	−	−	−	−	−
268	0.91	−	−	−	−	−	2.90(0.62)	−
270	0.57	−	−	−	−	−	−	−
274	−0.99	−	−	−	−	−	−1.90(0.46)^*b*^	−
361	0.41	0.30(0.16)^*b*^	0.40(0.16)^*b*^	0.15(0.56)^*b*^	0.77(0.24)^*b*^	−0.72(0.21)^*b*^	1.80(0.40)^*b*^	−
461	0.51	−	−	−	−	−	−	−
Parameter(s)		*λ*=0.050	*a* = 0.01	*λ*=0.0257	*a* = 0.1	$\begin{array}{c} \upsilon =10^{-3}\\ \tau =10^{-4} \end{array}$		
			*b* = 6		*α=0.05*			
CPU time(s)		25.56	1.31	1.67	1.90	20.64	54.70	8.84
true/false positive		11/2^*c*^	11/1^*c*^	6/4^*c*^	10/1^*c*^	9/0^*c*^	17/18^*c*^	8/25^*d*^

^*a*^The estimated marker effect is denoted by $\hat{\beta }$ and the standard deviation is denoted by $s_{\hat{\beta }}$.

^*b*^The estimated marker effect was obtained from a neighboring marker (≤ 20 cM) rather than from the marker with true effect.

^*c*^Number of effects with a *p*-value ≤ 0.05.

^*d*^Number of effects with a *p*-value ≤ 1.04×10^-4^ after Bonferroni correction was applied.

The average log likelihood (denoted as *logL*) from ten-fold cross validation was used to select the optimal value of the hyperparameter(s) in the EBLASSO-NE, the EBLASSO-NEG, the LASSO. Specifically, the dataset was first divided into 10 subsets. Nine subsets were used as the training data to estimate model parameters and the log likelihoods of the remaining testing data were calculated using the estimated parameters. This process was repeated ten times until every subset had been tested. The *logL* was the average of all the likelihoods obtained from 10 testing datasets.

For the EBLASSO-NE, we first calculated *λ*_max_ as described earlier. We then chose a set of values for *λ* decreasing from *λ*_max_ to 0.001 at a step of 0.35 on the logarithmic scale. Ten-fold cross validation using this set of values identified an optimal value denoted as *λ*_1._ We next zoomed in the interval of length 0.01 centering at *λ*_1_, and performed cross validation using ten more values equally spaced in the interval. This procedure identified the largest *logL* at the optimal *λ =* 0.050. A summary of results for several values of *λ* and the corresponding *logL* was given in Table 2. Using the optimal values of *λ*, the EBLASSO-NE detected 11 true and 2 false positive effects that have *p*-value ≤ 0.05. The estimated sizes of the true effects and their standard errors were given in Table 1.

**Table 2:** Cross-validations of the EBLASSO-NE, EBLASSO-NEG and LASSO for the simulation with only main effects

**Algorithm**	**Parameters**^***a***^	***logL *** **± *STE***^***b***^
	0.0011	−0.39 ± 0.03
	0.0022	−0.42 ± 0.03
	0.0447	−0.42 ± 0.04
EBLASSO-NE	0.0500	−0.36 ± 0.02^*c*^
	0.0631	−0.39 ± 0.02
	0.1259	−0.41 ± 0.03
	0.2512	−0.40 ± 0.01
	(−0.5,0.05)	−0.38 ± 0.03
	(0.01,0.05)	−0.37 ± 0.02
	(1,0.05)	−0.47 ± 0.02
EBLASSO-NEG	(0.01,5)	−0.39 ± 0.03
	(0.01,6)	–0.36 ± 0.02^*c*^
	(0.01,7)	−0.37 ± 0.02
	0.1037	−0.56 ± 0.02
	0.0516	−0.44 ± 0.03
LASSO	0.0257	−0.37 ± 0.04
	0.0128	−0.35 ± 0.05^*c*^
	0.0064	−0.36 ± 0.06

^*a*^Parameters are *λ* for EBLASSO-NE and LASSO, (*a*, *b*) for EBLASSO-NEG.

^*b*^The average log likelihood and standard error were obtained from ten-fold cross validation.

^*c*^The optimal log likelihood and corresponding parameter(s) chosen for comparison with other methods.

The optimal values of parameters *a* and *b* in the EBLASSO-NEG were obtained with cross validation in three steps. In the first step, *a* = *b* = 0.001, 0.01, 0.1, 1 were examined and a pair (*a*_1_, *b*_1_) corresponding to the largest *logL* was obtained. In the second step, *b* was fixed at *b*_1_ and *a* was chosen from the set [−0.5, -0.4, -0.3, -0.2, -0.1, -0.01, 0.01, 0.05, 0.1, 0.5, 1], which yielded an *a*_2_ corresponding to the largest *logL*. Note that when fixing one of the two parameters, the degree of shrinkage is a monotonic function of the other parameter. In the third step, *a* = *a*_2_ was fixed and *b* was varied from 0.01 to 10 with a step size of one for *b* > 1 and a step size of one on the logarithmic scale for *b* < 1. This three-step procedure identified the optimal pair of parameters that maximized *logL* at (*a*, *b*) = (0.01, 6). A summary of results for several pairs of *a* and *b* and the corresponding *logL* was given in Table 2. The whole dataset was then analyzed with the optimal parameters, which identified 11 true and 1 false positive effects that have *p*-value ≤ 0.05. The estimated sizes of true effects and their standard errors were depicted in Table 1.

For the LASSO-logistic regression, the cross validation procedure in the *glmnet* package [42] automatically identified a maximum value *λ*_max_ for *λ* that gave one nonzero effect and then *λ* was decreased from *λ*_max_ to *λ*_min_ = 0.001*λ*_max_ with a step size of [log(*λ*_max_) − log(*λ*_min_)]/100 on the logarithmic scale. The largest *λ* that yielded a *logL* within one standard error of the maximum *logL* was determined as the optimal value. This gave the optimal values *λ* = 0.0257. The whole dataset was then analyzed using the optimal *λ* to estimate QTL effects, which identified 40 markers with nonzero regression coefficients. The LASSO only estimates regression coefficients without giving a confident interval or a *p*-value for the estimates. If we counted all nonzero coefficients as detected effects and considered them as one effect if they were in 20 cM from a QTL, the LASSO yielded 14 true positive effects and 12 false positive effects. Moreover, the LASSO typically gives a biased estimate of non-zero coefficient toward zero. To reduce the false positive rate and the bias, we refitted an ordinary logistic regression model with the markers selected by the LASSO. Among those markers with a *p*-value ≤ 0.05 in the refitted model, 6 markers were true positive effects 4 were false positive effects. A summary of results from cross validation for the EBLASSO-NE, the EBLASSO-NEG and LASSO was given in Table 2. The estimated sizes of true effects and their standard errors were depicted along with all 20 true effects in Table 1.

The HyperLasso employed the same Bayesian NEG hierarchical prior for the marker effects and estimated the posterior modes using a numerical algorithm [25]. Hoggart *et al.* did not propose a cross validation procedure to determine the values of *a* and *b* but suggested a range from 0.01 to 10 for *a* and gave a formula to calculate *b* from the level of the type-I error controlled at *α*. In our simulations, we used three values for *a* and four values for *α* as listed in Table 3. The values of *b* calculated from *α* using the method in [25] is also included in Table 3. Similar to the LASSO, the HyperLasso outputs a point estimate of ***β*** without a confidence interval or a *p*-value. Therefore, we refitted the markers selected by the HyperLasso with ordinary logistic regression and identified markers with a *p*-values ≤ 0.05 as QTLs. The number of effects identified with different values of *a* and *b* are presented in Table 3. The best results in Table 3 include 10 true and 1 false positive effects. We would emphasize here that these best results may not be achievable in the analysis of real data because the optimal values of *a* and *b* cannot be determined. The estimated sizes of true effects and their standard errors for the best results in Table 3 were depicted along with all 20 true effects in Table 1.

**Table 3:** Summary of results of the HyperLasso for the simulated data with only main effects

**Parameters**			**True/False positive effects**^***a***^
**Shape *a***	**Inverse scale *b***	**Type I error *α***	
0.1	1.7 × 10^-3^	0.05	10/1^*b*^
0.05	1.5 × 10^-3^		9/2
0.01	1.4 × 10^-3^		10/2
0.1	9.8 × 10^-4^	0.01	9/1
0.05	8.8 × 10^-4^		9/1
0.01	7.9 × 10^-4^		9/1
0.1	5.2 × 10^-4^	$\frac{0.05}{481}$	8/1
0.05	4.7 × 10^-4^		8/1
0.01	4.2 × 10^-4^		8/1
0.1	3.6 × 10^-4^	$\frac{0.01}{481}$	7/0
0.05	3.2 × 10^-4^		7/0
0.01	2.9 × 10^-4^		7/0

^*a*^Effects with *p*-value ≤ 0.05 were considered as significant different from zero.

^*b*^The optimal results chosen for comparison with other methods.

The BhGLM method [26] employed a two-level Bayesian hierarchical prior for the marker effects: the *i*th entry of ***β*** follows a normal distribution *N*(0, *σ*_*i*_^2^) and *σ*_*i*_^2^ obeys an inverse- *χ*^2^ distribution *Inv* - *χ*^2^(*υ*_*i*_, *τ*_*i*_^2^), and it used the EM algorithm to estimate the posterior mode of the Bayesian QTL model. The default value for hyperparameters *ν*_*i*_ and *τ*_*i*_ are 0.01 and 10^-4^, respectively. The variance of regression coefficients in BhGLM method was treated as missing data and estimated in the E-step of the EM algorithm. The *p*-value of each nonzero effect was calculated from the *t*-distribution of one degree of freedom using the *t*-statisrics calculated from the estimated regression coefficient and corresponding variance. We examined 15 different pairs of values for *ν*_*i*_ and *τ*_*i*_ as listed in Table 4. The values *ν*_*i*_ = 10^-3^, 10^-4^ and 10^-5^ all gave the best result which includes 9 true and 0 false positive effects. The corresponding estimated sizes of the true effects and their standard errors were depicted in Table 1.

**Table 4:** Summary of results of the BhGLM for the simulated data with only main effects

**Parameters**		**True/False positive effects**^***a***^
***ν***	***τ***	
10^-5^		9/0
10^-4^		9/0
10^-3^	10^-5^	9/0
10^-2^		9/0
10^-1^		9/0
10^-5^		9/0
10^-4^		9/0
10^-3^	10^-4^	9/0^*b*^
10^-2^		9/0
10^-1^		9/0
10^-5^		9/0
10^-4^		9/0
10^-3^	10^-3^	9/0
10^-2^		9/0
10^-1^		9/0

^*a*^Effects with *p*-value ≤ 0.05 were considered as significant different from zero.

^*b*^The optimal results chosen for comparison with other methods.

The RVM for classification [33] assumed a uniform prior for the variance of regression coefficients, and thus it did not involve parameter selection. We utilized the MATLAB implementation of the RVM from the authors [33] to analyze the datasets and identified 35 nonzero effects with a *p*-value ≤ 0.05. Among these effects, we have 17 true and 18 false positives. The estimated sizes of true effects and their standard errors were depicted in Table 1. The false positive rates were much higher that the EBLASSO-NEG, EBLASSO-NE, LASSO, and HyperLasso. When we reduced the number of false positive effects to three, at a level slightly higher than that of our EBLASSO-NEG and EBLASSO-NE, by reducing the cutoff for the *p*-value, we obtained 10 true positive effects, which were smaller than the number of true effects identified with our EBLASSO-NEG and EBLASSO-NE.

Single QTL mapping with logistic regression was performed using the *glm* procedure in R [41]. After Bonferroni correction, effects with a *p*-value ≤ 0.05/481 = 1.04 × 10^-4^ were considered as significant, which identified 8 true and 25 false positive effects. The estimated sizes of true effects and their standard errors were depicted in Table 1. The small *p*-value cutoff used by Bonferroni correction was expected to yield a small false positive rate. However, the single QTL mapping method with Bonferroni correction still gave much more false positive effects than other methods. If we had used another popular permutation technique for multiple test correction, we effectively employed a larger *p*-value cutoff. Although this could increase power of detection, it would also increase the false positive rate. To see this, we increased the cutoff for the *p*-value to 6 × 10^-4^ so that the number of true positive effects detected was increased to 11, at a level same as that of our EBLASSO-NEG and EBLASSO-NE methods, but then the number of false positive effects was increased to 27.

As shown in Table 1, the EBLASSO-NE and the EBLASSO-NEG identified more true effects than other four methods except RVM, and yielded a number of false positive effects comparable to those of the LASSO, the HyperLasso and the BhGLM but much smaller than those of the RVM and the single-QTL mapping method. Note that the false negative rate can be easily calculated from 20 simulated true effects and the true positive effects detected by each method. While the EBLASSO-NE and the EBLASSO-NEG offered similar performance in terms of power of detection and the false positive rate, several true effects were detected by either of them, which implies that the power of detection could be improved if the results of two methods were combined. Similar observations can be seen from the simulation results for two more independent replicates described in Additional file 1: Table S1 and Table S2.

It is well known that the LASSO typically selects only one variable among a set of highly correlated variables. This phenomenon is indeed observed from the results in Tables 1, Additional file 1: Tables S1 and S2 for two pairs of highly correlated markers, (72,73) and (181,182). It turns out that all methods compared except the single QTL mapping method have the same problem, although the problem with the EBLASSO-NE tends to be less severe. The LASSO is also known to bias the regression coefficients toward zero. This is observed from the results in Table 1, Additional file 1: Tables S1 and S2. Since the EBLASSO-NE uses the same prior distribution as the LASSO, it exhibits the same trend. However, the RVM inflated all the detected effects likely due to its small degree of shrinkage. On the other hand, the EBLASSO-NEG, the HyperLasso and the BhGLM tend to detect only one of the two highly correlated effects with an inflated effect size.

All simulations were performed on a personal computer (PC) with a 3.4 GHz Intel PentiumD CPU and 2Gb memory running Windows XP, except that the HyperLasso was ran on an IBM BladeCenter cluster including computing nodes with 2.6 GHz Xeon or 2.2 GHz Opteron CPU running Linux. The speeds of the EBLASSO-NEG, the LASSO and the HyperLasso are comparable and faster than the other methods. The speeds of the EBLASSO-NE, the BhGLM and the single-QTL mapping method are comparable.

#### Simulation results for the model with main and epistatic effects

In the second simulation setup, 10 main and 10 epistatic effects were simulated. The genotypes of *m* = 481 markers of *n* = 1,000 individuals were generated using the procedure described earlier. Locations and effects of the QTLs and QTL pairs are shown in Table 5. Some of the markers had only main or epistatic effect, while the others had both main and epistatic effects. The status of the binary trait of each individual was generated using the logistic regression model as described in the first simulation setup. The QTL model contained a total of $k=1+481+\left(\begin{array}{c} 481\\ 2 \end{array}\right)=115,922$ possible effects, a number about 116 times of the sample size, and the design matrix **X** was of size 1000 × 115,921. QTL mapping was performed with all methods described earlier. However, the BhGLM method did not converge and the RVM ran out of memory due to a large number of nonzero effects included in the model, and thus, they did not yield any result.

**Table 5:** True and estimated effects for the simulated data with main and epistatic effects

**locus *i***	**locus *j***	**True *β***	**EBLASSO- NE**$\hat{\beta }{\left(s_{\hat{\beta }}\right)}^{a}$	**EBLASSO-NEG**$\hat{\beta }{\left(s_{\hat{\beta }}\right)}^{a}$	**LASSO**$\hat{\beta }{\left(s_{\hat{\beta }}\right)}^{a}$	**HyperLasso**$\hat{\beta }{\left(s_{\hat{\beta }}\right)}^{a}$	**Two-locus test**$\hat{\beta }{\left(s_{\hat{\beta }}\right)}^{a}$
11	11	1.99	0.83(0.12)	1.66(0.19)	0.72(0.65)	2.21(0.25)	0.88(0.10)
26	26	1.81	0.46(0.11)	1.42(0.18)	0.39(0.55)	1.73(0.23)	0.56(0.09)
42	42	−1.28	−0.36(0.11)	−0.87(0.20)	−	−1.59(0.21)^*b*^	−
48	48	−0.91	−0.19(0.09)^*b*^	−0.68(0.19)^*b*^	−0.14(0.55)^*b*^	−	−
72	72	1.28	1.01(0.16)	2.53(0.20)	0.92(1.18)	3.17(0.27)	1.08(0.10)
73	73	1.81	0.40(0.14)	−	−	−	1.04(0.10)
182	182	2.19	0.50(0.14)	1.57(0.26)	0.51(0.96)	2.03(0.30)	1.23(0.10)
185	185	1.29	0.69(0.14)	1.49(0.26)	0.57(0.91)	1.88(0.30)	1.23(0.10)
262	262	−1.28	−0.24(0.09)	−0.70(0.15)	−0.15(0.46)	−0.78(0.19)^*b*^	−
268	268	0.91	−	−	−	−	−
5	6	1.28	0.42(0.13)	1.11(0.22)	0.40(0.63)	1.63(0.28)	−
6	39	1.29	0.38(0.15)^*b*^	1.37(0.23)^*b*^	0.15(1.16)^*b*^	1.28(0.35)^*b*^	−
42	220	1.99	0.23(0.13)	1.99(0.25)^*b*^	−	2.47(0.32)	0.77(0.14)
81	200	−1.28	−0.36(0.13)^*b*^	−1.02(0.22)^*b*^	−0.15(1.42)^*b*^	−1.22(0.27)^*b*^	−
87	164	1.81	0.44(0.17)	1.73(0.25)	0.24(1.44)	2.15(0.32)^*b*^	−
87	322	2.19	0.90(0.15)	2.10(0.25)	0.74(0.66)	2.44(0.30)	0.79(0.13)
118	278	−1.28	−0.29(0.12)	−0.76(0.20)	−0.19(1.29)	−0.99(0.26)	−
328	404	−0.99	−0.21(0.12)^*b*^	−	−0.15(0.73)^*b*^	−1.15(0.30)^*b*^	−
373	400	−0.91	−0.22(0.12)^*b*^	−1.12(0.22)^*b*^	−0.19(0.87)	−1.23(0.27)	−
431	439	1.81	0.24(0.13)	1.37(0.24)	−	1.58(0.29)^*b*^	−
Parameter(s)			*λ* = 0.1600	*a* = −0.2	*λ*=0.0254	*a* = 0.1	
				*b* = 0.1		α=0.01	
CPU time(s)			2037.4	268.6	62.7	1094.6	2936.0
True/False positive			19/5^*c*^	17/4^*c*^	15/26^*c*^	17/7^*c*^	8/18^*d*^

^*a*^The estimated marker effect is denoted by $\hat{\beta }$ and the standard deviation is denoted by $s_{\hat{\beta }}$.

^*b*^The estimated marker effect was obtained from a neighboring marker (≤ 20 cM) rather than from the marker with true effect.

^*c*^Number of effects with *p*-value ≤ 0.05.

^*d*^Number of effects with a *p*-value ≤ 4.31×10^-7^ after Bonferroni correction was applied.

The same cross-validation procedures described earlier were performed to choose the optimal values of the hyperparameters for the EBLASSO-NE, the EBLASSO-NEG and LASSO, and the results for several values of hyperparameters are presented in Table 6. Optimal values of the hyperparameters were then used to analyze the whole dataset. The number of true and false positive effects identified and the estimated effect sizes of the detected true effects are given in Table 5.

**Table 6:** Cross-validations of the EBLASSO-NE, EBLASSO-NEG and LASSO for the simulation with main and epistatic effects

**Algorithm**	**Parameters**^***a***^	***logL *** **± *STE***^***b***^
	0.0631	−0.44 ± 0.04
	0.0891	−0.41 ± 0.04
	0.1259	−0.39 ± 0.03
EBLASSO-NE	0.1600	−0.37 ± 0.01^*c*^
	0.1778	−0.42 ± 0.04
	0.2512	−0.53 ± 0.04
	0.3548	−0.47 ± 0.04
	(−0.4,0.05)	−0.40 ± 0.05
	(−0.2,0.05)	−0.20 ± 0.05
	(−0.1,0.05)	−0.10 ± 0.05
EBLASSO-NEG	(−0.2,0.01)	−0.35 ± 0.02
	(−0.2,0.1)	−0.33 ± 0.02^*c*^
	(−0.2,0.5)	−0.35 ± 0.02
	0.1027	−0.57 ± 0.01
	0.0511	−0.47 ± 0.02
LASSO	0.0254	−0.37 ± 0.02^*c*^
	0.0127	−0.37 ± 0.03
	0.0063	−0.39 ± 0.04

^*a*^Parameters are *λ* for EBLASSO-NE and LASSO, (*a*, *b*) for EBLASSO-NEG.

^*b*^The average log likelihood and standard error were obtained from ten-fold cross validation.

^*c*^The optimal log likelihood and corresponding parameter(s) chosen for comparison with other methods.

For the HyperLasso, we again examined 12 pairs of values for hyperparameters *a* and *b* as listed in Table 7, and identified *a* = 0.1 and *b* = 4.9 × 10^-4^ as the optimal values that gave best tradeoff between the true and false positive effects. We also used a two-locus logistic regression model logit(*p*_*i*_) = *β*_0_ + *β*_1_*x*_*i*_ + *β*_2_*x*_*j*_ + *β*_3_*x*_*i*_ ⋅ *x*_*j*_, *i* = 1, ⋯, m-1, *j* > *i*, to test the epistatic effect of locus *i* and *j*. The logistic regression model was fitted with the *glm* procedure in R [41]. Effects with a *p*-value ≤ 0.05/*k* = 4.31 × 10^-7^ were considered as significant. Detailed QTL mapping results for the HyperLasso and the two-locus test are also given in Table 5.

**Table 7:** Summary of results of the HyperLasso for the simulated data with main and epistatic effects

**Parameters**			**True/False positive effects**^***a***^
**Shape** ***a***	**Inverse scale *b***	**Type I error *α***	
0.1	8.5 × 10^-4^	0.05	17/18
0.05	7.6 × 10^-4^		19/18
0.01	6.8 × 10^-4^		19/19
0.1	4.9 × 10^-4^	0.01	17/7^*b*^
0.05	4.4 × 10^-4^		17/8
0.01	3.9 × 10^-4^		17/8
0.1	1.3 × 10^-4^	$\frac{0.05}{115921}$	5/0
0.05	1.1 × 10^-4^		5/0
0.01	1.0 × 10^-4^		8/0
0.1	1.1 × 10^-4^	$\frac{0.01}{115921}$	5/0
0.05	1.0 × 10^-4^		5/0
0.01	0.9 × 10^-4^		5/0

^*a*^Effects with *p*-value ≤ 0.05 were considered as significant different from zero.

^*b*^The optimal results chosen for comparison with other methods.

As shown in Table 5, the EBLASSO-NE and the EBLASSO-NEG detected the same or a larger number of true effects but a smaller number of false positive than the other methods, which clearly demonstrates that our EBLASSO-NE and EBLASSO-NEG offer the best overall performance in terms of power of detection and the false positive rate. The LASSO is the fastest, while the EBLASSO-NEG and the HyperLasso are the second and the third fastest. Similar observations were obtained from two more independent simulations, as shown in the results for replicates 2 and 3 whose details were presented in the Additional file 1.

### Real data analysis

We used a mouse data published by Masinde *et al.*[43] as an example to test our methods. The trait was wound healing speed of mice measured as a binary trait, fast healer denoted by 1 and slow healer denoted by 0. There were 633 F_2_ mice derived from the cross of a faster healer inbred line (MRL/MPj) and a slow healer inbred line (SJL/J). At age 3 weeks, each F_2_ mouse was punched a 2-mm hole in the lower cartilaginous part of each ear using a metal ear puncher. The fast healer mice completely healed in 21 days after ear punch (complete closure of the holes) while the slow healer mice remained open for the holes after 21 days of ear punch. Some of the F_2_ mice healed partially and these mice were phenotypically coded as 1 if the holes were < 0.7 mm and 0 if the holes were > 0.7 mm. This dataset consisted of the genotypes of 119 markers across the mouse genome from 633 samples. Samples with more than 10% of missing markers or with missing phenotype were removed, resulting in a 532 × 119 genotype matrix with 3.28% missing values. These missing genotypes were inferred from neighboring markers. Total number of possible effects is *k* = 7141.

We carried out QTL mapping for this dataset using the EBLASSO-NE, the EBLASSO-NEG, the LASSO and the HyperLasso, since simulation results presented earlier show these four methods offer better performance than the other methods. Ten-fold cross validation for the EBLASSO-NE, the EBLASSO-NEG and the LASSO were performed with the same procedures used in simulation studies to obtain optimal values of the hyperparameters. For the EBLASSO-NE, *λ* = 0.4 was determined as the optimal value, which resulted in 7 main and 4 epistatic effects with a *p*-value ≤ 0.05. For the EBLASSO-NEG, (*a, b*) = (0.01, 0.5) were the optimal values which resulted in 4 main and 4 epistatic effects with a *p*-value ≤ 0.05. For the LASSO, *λ* = 0.0715 was the optimal value*,* which yielded 13 non-zero effects. Refitting an ordinary logistic regression model with these 13 effects to the data identified 7 main and 3 epistatic effects with a *p*-value ≤ 0.05. When *α* = 0.05/*k* and 0.01/*k*, the HyperLasso only identified 1 or 2 main effects; when *α* = 0.05, it identified more than 40 effects. On the other hand, when *a* = 0.1 and α = 0.01, it identified 8 main and 17 epistatic effects, all having a *p*-value ≤ 0.05, which seemed a more reasonable result than the ones obtained with other values of *a* and *α*. Therefore, these 25 effects were regarded as the effects identified by the HyperLasso. The effects identified by at least 3 of the 4 methods are listed in Table 8, which contain 7 main and 3 epistatic effects.

**Table 8:** Results for the real data obtained with EBLASSO-NE, EBLASSO-NEG, LASSO and HyperLasso

**Marker/Marker pair**^***a ***^**IDs**	**Position (Chr,cM)**	**EBLASSO-NE**$\hat{\beta }{\left(s_{\hat{\beta }}\right)}^{b}$	**EBLASSO-NEG**$\hat{\beta }{\left(s_{\hat{\beta }}\right)}^{b}$	**LASSO**$\hat{\beta }{\left(s_{\hat{\beta }}\right)}^{b}$	**HyperLasso**$\hat{\beta }{\left(s_{\hat{\beta }}\right)}^{b}$
D1mit334^*d*^	(1,49.2)	−0.15(0.28)	−	−0.37(0.19)	−0.80(0.18)
D3mit217^*d*^	(3,43.7)	−0.20(0.30)	−0.62(0.13)	−0.42(0.20)	−
D4mit214^*d*^	(4,21.9)	−0.24(0.30)	−	−0.46(0.16)	−0.81(0.20)
D6mit261^*d*^	(6,29.5)	−0.18(0.29)	−0.42(0.12)	−0.56(0.15)	−0.78(0.18)
D9mit270^*d*^	(9,41.5)	−0.25(0.31)	−0.72(0.13)	−0.39(0.23)	−0.57(0.24)
D9mit182	(9,53.6)	−0.25(0.32)	−	−0.51(0.20)	−0.80(0.26)
D13mit228^*d*^	(13,45.9)	−0.12(0.27)^*c*^	−0.40(0.12)^*c*^	0.38(0.20)	0.91(0.19)
(D1mit19;D17mit176)	(1,37.2;17,12.0)	0.32(0.37)	0.60(0.19)	0.89(0.24)	0.92(0.30)
(D4mit31^*d*^;Dxmit208)	(4,50.3;20,18.6)	0.19(0.33)	0.72(0.18)	0.71(0.23)	0.69(0.29)
(D7mit246;D11mit242)	(7,12.0;11,31.9)	0.20(0.33)	0.48(0.16)	−	0.71(0.25)

^*a*^Paired markers in parenthesis are markers involved in an epistatic effect. Only effects detected by at least three of the four algorithms are shown. All effects listed have a *p-*value ≤ 0.05.

^*b*^Parameters are *λ* = 0.4 for EBLASSO-NE, (*a*, *b*) *=* (0.01, 0.5) for EBLASSO-NEG, *λ* = 0.0715 for LASSO and (*a*,α) = (0.1, 0.01) for HyperLasso. The estimated marker effect is denoted by $\hat{\beta }$ and the standard deviation is denoted by $s_{\hat{\beta }}$.

^*c*^The estimated marker effect was obtained from a neighboring marker D13mit35 (59.0 cM).

^*d*^Markers identified previously by Masinde *et al.*[41].

Masinde *et al.*[43] previously identified 10 main effects using a single-QTL mapping method, and 8 epistatic effects using two-way ANOVA. Among the 7 main effects and 3 epistatic effects identified in our analysis, 6 of the main effects are identical to those identified by Masinde *et al.* but all 3 epistatic effects are different from the ones identified by Masinde *et al.* One of the markers involved in an epistatic effect (D4mit31) identified in our analysis was a main effect identified by Masinde *et al.* Since our QTL model considers the main and epistatic effects jointly, it may account for both effects more reliably, comparing with the single-QTL mapping approach and the two way ANOVA used by Masinde *et al.* Therefore, the three epistatic effects identified in our analysis may be novel effects that are worth further experimental investigation.

Some of the identified QTLs are in positions close to the genes that are up-regulated in expression profiles obtained during the inflammation stage of wound healing [44]. Loci D3mit217 and D9mit270 were identified as main effects in both our analysis and the study of Masinde *et al.* It turns out that D3mit217 (chr3, 34.7 cM) is close to genes *calgranulin A* (chr3, 43.6 cM), *CD53* (chr3, 50.5 cM), and *small proline-rich protein 1A* (chr3, 45.2 cM), and that D9mit270 (chr9, 41.5 cM) is close to gene *annexin A2* (chr9, 37.0 cM). Locus D9mit182 was identified as a main effect in our study but not identified as an effect in the study of Masinde *et al.* It was found that D9mit182 (chr9, 53.6 cM) is close to *chemokine receptor 2* (chr9, 71.9 cM). Among the loci in the three epistatic effects we identified, D11mit242 (chr11, 31.7 cM) is close to *chemokine (C-C motif) ligase 4* (chr11, 47.6 cM) and *chemokine (C-C motif) ligase 6* (chr11, 41.5 cM). Genes related to growth factors are known to play an important role in wound healing [43,45]. D7mit246 and D17mit176 are involved in the epistatic effects we identified; and D7mit246 is 5.0 cM away from the *fibroblast growth factor receptor 2* (*FGFR 2*), and D17mit176 is 12.2 cM away from the *vascular endothelial growth factor* (*VEGF*).

## Discussion

Our EBLASSO-NEG algorithm is based on a Bayesian logistic regression model that uses the same three-level hierarchical prior for the regression coefficients as the one used in the Bayesian LASSO linear regression model [23,30,31], the Bayesian hyper-Lasso linear regression model [35] and the HyperLasso logistic regression model [25]. The HyperLasso of Hoggart *et al.*[25] uses a numerical algorithm to estimate the mode of the posterior distribution, whereas our EBLASSO-NEG first estimates the variance of the regression coefficients and then find an approximation of the posterior distribution of the regression coefficients based on the estimated variance. As shown in our simulation study, our EBLASSO-NEG offers better performance than the HyperLasso of Hoggart *et al.* in terms of power of detection, false positive rate and speed, especially when the number of possible effects is very large in QTL models with both main and epistatic effects.

The LASSO-logistic regression was applied to GWAS to identify genomic loci associated with complex disease [28,29], and it can be directly employed in QTL mapping for binary traits as shown in our simulation study and real data analysis. The LASSO-logistic regression particularly implemented with the *glmnet* algorithm [42] is very efficient. Our EBLASSO-NE algorithm and the LASSO-logistic regression essentially employ the same two-level prior for the regression coefficients. However, the major difference is that the LASSO-logistic regression estimates the mode of the posterior distribution, whereas our EBLASSO-NE algorithm first estimates the variances of the regression coefficients and then finds the posterior distributions of the regression coefficients. In our simulation study, we demonstrated that both our EBLASSO-NE and EBLASSO-NEG algorithms outperform the LASSO-logistic regression in terms of power of detection and false positive rate, although their speed is lower than that of the LASSO. The good performance of our EBLASSO-NE and EBLASSO-NEG may be due to the fact that our model inference using the variance and posterior distribution of the regression coefficients provides more information than the point estimation of the regression coefficients yielded by the LASSO-logistic regression.

Another prior distribution commonly employed in Bayesian shrinkage is the mixture of normal and inverse-*χ*^2^ distributions as used in the Bayesian linear regression model for continuous traits [23,24,46] and the generalized linear model for continuous or binary traits in the BhGLM method [26]. The BhGLM method uses the EM algorithm to estimate the mode of the posterior distribution treating the variance of regression coefficients as missing data. As shown in our simulations for QTL models with only main effects, our EBLASSO-NEG and EBLASSO-NE offer power of detection better than and a false positive rate comparable to the BhGLM method. The speed of the EBLASSO-NEG is much higher than that of the BhGLM method, while the speed of the EBLASSO-NE is comparable to that of the BhGLM method. However, the BhGLM method was not able to handle a QTL model with 115,921 variables and 1,000 samples in our simulation, whereas our EBLASSO-NEG and EBLASSO-NE completed the analysis of this model within 5 min and 35 min, respectively.

Our EBLASSO-NEG and EBLASSO-NE use the same Laplace’s method originally proposed in [38] as the one used in the RVM for classification [32,33]. However the prior distributions used by three methods are different. Although both the uniform prior and the inverse gamma prior for the variance of regression coefficients were considered in [32,33], the more efficient RVM algorithm [33] employs the uniform prior. The uniform prior does not have any hyperparameter and lacks flexibility of adjusting the degree of shrinkage that our EBLASSO-NEG and EBLASSO-NE enjoy. The uniform prior of the RVM causes at least two problems. First, it often does not provide sufficient degree of shrinkage in multiple QTL mapping that includes a very large number of possible effects, which results in a large number of false positive, as observed in our simulations in this paper and in the simulation study for QTL mapping for continuous traits in [30,46]. Second, because of the relatively low degree of shrinkage, the regression model usually contains a relatively large number of nonzero regression coefficients, which reduces the speed of the algorithm, as seen in our simulations.

Our EBLASSO-NEG and EBLASSO-NE estimate the variance of regression coefficients iteratively. In each iteration, Laplace’s method is first used to obtain an approximation of the posterior distribution, which results in an equivalent linear regression model. Then, the EBLASSO-NEG uses the EBLASSO algorithm we developed in [30] to estimate the variance. Therefore, the EBLASSO-NEG essentially is a combination of Laplace’s method and the EBLASSO algorithm. However, the EBLASSO does not consider the prior of the EBLASSO-NE, and thus, we derive a novel formula in (12) and modify the EBLASSO algorithm to estimate the variance for EBLASSO-NE. The EBLASSO-NEG has two hyperparameters, whereas the EBLASSO-NE has only one hyperparameter, which simplifies cross-validation for selecting the optimal value of the hyperparameter. Moreover, simulation studies demonstrated that the EBLASSO-NE identified some QTLs that were not detected by the EBLASSO-NEG, suggesting that combination of the two methods can lead to increased detection power.

The full Bayesian methods [11-13,15,47] use the threshold model that employs a latent liability variable to link the binary trait with the QTLs and then apply MCMC simulation to infer the model. It is well known that MCMC simulation requires very large computation for the model with a relatively large number of variables. Therefore, these fully Bayesian methods may not be computationally efficient for QTL mapping with both main and epistatic effects from a relatively large number of QTLs.

The MIM methods [17,18] for mapping binary trait loci either does not employ any variable selection technique [17] or uses step-wise logistic regression and BIC to select variables [18]. Hence, it is difficult for them to deal with a QTL model with a relatively large number of loci and epistatic effects due to their demanding computation burden and possible large false discovery rate. The probability model in [19] for binary trait mapping entails a model space exponentially increasing with the number of loci. Although the BIC is used for model selection, its computational complexity increases dramatically with the number of loci.

We demonstrated that our EBLASSO-NE and EBLASSO-NEG can easily handle a model with 115,922 variables. If much more variables are involved, in e.g. GWAS or QTL mapping with high order interactions, our methods can be combined with a variable screening method such as the sure independence screening (SIS) [48,49] to facilitate computation. It is not difficult to extend our EBLASSO-NE and EBLASSO-NEG algorithms to QTL mapping for ordinal traits. We can first apply Laplace’s method to get an approximately equivalent linear model by deriving the gradient and the Hessian matrix of the posterior distribution and then apply our efficient EBLASSO algorithm to infer the linear model. Since the multinomial logistic regression model includes more variables than the binary logistic regression model, it will require more computation.

## Conclusions

We have developed two algorithms named EBLASSO-NEG and EBLASSO-NE for the inference of Bayesian logistic regression models for multiple QTL mapping. While the EBLASSO-NEG is an extension of the EBLASSO [30] to handle binary traits, the simulations demonstrate that the EBLASSO-NEG algorithm provides superior performance in terms of power of detection and false positive rate, comparing with five other existing methods. Moreover, the EBLASSO-NEG is faster than four other methods tested but only slower than the LASSO algorithm. The hierarchical prior in the EBLASSO-NE in this paper was not considered in the EBLASSO [30]. Here we derive a novel formula given in equation (12) to estimate the variance of regression coefficients. Our simulations show that the EBLASSO-NE provides comparable or better power of detection and false positive rate comparing with five other existing methods, and the power of detection could be improved if the results are combined with that of the EBLASSO-NEG. In summary, our EBLASSO-NE and EBLASSO-NEG algorithms provide an efficient tool for QTL mapping for binary traits involving a large number of both main and epistatic effects, and they can be extended to QTL mapping for ordinal traits.

## Abbreviations

AIC: Akaike information criterion; BIC: Bayesian information criterion; BhGLM: Bayesian hierarchical generalized linear model; BLASSO: Bayesian LASSO; EB: Empirical Bayes; EBLASSO: Empirical Bayesian LASSO; EM: Expectation-maximization; GWAS: Genome-wide association study; LASSO: Least absolute shrinkage and selection operator; MAP: Maximum *a posterior*; MCMC: Markov Chain Monte Carlo; MIM: Multiple-interval mapping; NE: Normal exponential prior; NEG: Normal exponential gamma prior; PC: Personal computer; QTL: Quantitative trait locus; RVM: Relevant vector machine.

## Competing interests

The authors declare that they have no competing interests.

## Authors’ contributions

AH participated in the design of the algorithms, developed the computer programs, performed the simulations and data analyses, and drafted the manuscript. SX participated in the development of the algorithms, designed simulation study, participated in the data analyses and helped to draft the manuscript. XC conceived of and coordinated the study, developed the algorithms, participated in the data analyses and drafted the manuscript. All authors read and approved the final manuscript.

## Supplementary Material

Additional file 1 **Derivation of equation (12).** Replicates 2 and 3 for the simulations with only main effects. Click here for file
