# Supplementary material for: Empirical Bayesian LASSO-logistic regression for multiple binary trait locus mapping
Source: BMC Genet. 2013 Feb 15;14:5. doi: 10.1186/1471-2156-14-5 (PMC3771412; doi:10.1186/1471-2156-14-5)
Supplement: Additional file 1 — Derivation of equation (12). Replicates 2 and 3 for the simulations with only main effects. [file 1471-2156-14-5-S1.pdf]

Additional file

Derivation of equation (12)

First we note that $\lim_{\alpha_i \to 0} L(\alpha_i) = -\infty$ and $\lim_{\alpha_i \to \infty} L(\alpha_i) = 0$. The derivative of $L(\alpha_i)$ is given by:

$$\frac{\partial L}{\partial \alpha_i} = \frac{(s_i - q_i^2 + 2\lambda)\alpha_i^2 + (s_i^2 + 4\lambda s_i)\alpha_i + 2\lambda s_i^2}{2\alpha_i^2(\alpha_i + s_i)^2}.$$

Let us write the numerator of the derivative as $J(\alpha_i) = (s_i - q_i^2 + 2\lambda)\alpha_i^2 + (s_i + 4\lambda)s_i\alpha_i + 2\lambda s_i^2$, and define $\Delta = s_i^2 + 8\lambda q_i^2$. Since $\lambda > 0$, we have $\Delta \geq 0$, which implies that $J(\alpha_i)$ = 0 have two roots:

$r_1 = \frac{-(s_i + 4\lambda) - \sqrt{\Delta}}{2(s_i - q_i^2 + 2\lambda)} \cdot s_i$ and $r_2 = \frac{-(s_i + 4\lambda) + \sqrt{\Delta}}{2(s_i - q_i^2 + 2\lambda)} \cdot s_i$. We consider the following three cases:

Case 1: $s_i - q_i^2 + 2\lambda > 0$

We have $r_1 < 0$ and $r_2 < 0$ since $s_i > 0$ and $\lambda > 0$. Therefore, $\partial L / \partial \alpha_i > 0$ for $\alpha_i > 0$ and $L(\alpha_i)$ is an increasing function of α_i. This implies that $L(\alpha_i)$ is maximized at $\alpha_i^* = \infty$.

Case 2: $s_i - q_i^2 + 2\lambda = 0$

In this case, we have $J(\alpha_i) = (s_i + 4\lambda)s_i\alpha_i + 2\lambda s_i^2$. It is clear that $J(\alpha_i) > 0$ for $\alpha_i > 0$. Hence $\partial L / \partial \alpha_i > 0$ and $L(\alpha_i)$ is an increasing function of α_i. Then $L(\alpha_i)$ is maximized at $\alpha_i^* = \infty$.

Case 3: $s_i - q_i^2 + 2\lambda < 0$

We have $r_1 > 0$ and $r_2 < 0$. Therefore, $\partial L / \partial \alpha_i > 0$ for $0 < \alpha_i < r_1$, $\partial L / \partial \alpha_i = 0$ for $\alpha_i = r_1$, and $\partial L / \partial \alpha_i < 0$ for $\alpha_i > r_1$. This implies that $L(\alpha_i)$ is maximized at $\alpha_i^* = r_1$ and $L(\alpha_i^*) > 0$ since $\lim_{\alpha_i \to \infty} L(\alpha_i) = 0$.

Summarizing the results in three cases, we obtain α_i^* given in (12).

Replicates 2 and 3 for the simulations with only main effects

The genotypes of m = 481 markers of n = 500 individuals were generated independently for Replicate 2 and 3 using the procedure described in the main text. Twenty QTLs and their IDs and effect sizes were the same as those of Replicate 1 given in Table 1. The two replicates were analyzed with the EBLASSO-NE, EBLASSO-NEG, LASSO, HyperLasso, BhGLM, RVM as well as the single QTL method.

The EBLASSO-NE method: The two-step procedure for the EBLASSO-NE both identified the optimal values λ = 0.0224 for replicates 2 and 3. A summary of results for several values of λ and the corresponding *logL* is given in Table S3. Using the optimal values of λ, the EBLASSO-NE detected 13 true and 1 false, and 12 true and 0 false positive effects that have p-value $\leq$ 0.05 for replicates 2 and 3. The estimated sizes of the true effects and their standard errors for the two replicates are given in Tables S1 and S2, respectively.

The EBLASSO-NEG method: The three-step procedure for the EBLASSO-NEG identified the optimal pair of parameters that maximized *logL* at (a, b) = (-0.5, 10) and (-0.4, 6) for the replicates 2 and 3, respectively. A summary of results for several pairs of a and b and the corresponding *logL* is given in Table S3. The whole dataset was then analyzed with the optimal parameters, which identified 13 true and 2 false positive effects, 12 true and 2 false positive effects that have p-value $\leq$ 0.05 for the two replicates, respectively. The estimated sizes of the true effects and their standard errors are depicted in Tables S1and S2 respectively for the two replicates.

The LASSO method: Cross validation identified the optimal values λ = 0.0197 and 0.0213 for replicates 2 and 3, respectively. The whole dataset was then analyzed using the optimal λ to estimate QTL effects, which identified 38 and 35markers with nonzero regression coefficients

for the two replicates, respectively. After refitted with an ordinary logistic regression model, 9 and 11 true positive effects, 4 and 2 false positive effects were among those markers with a *p*-value ≤ 0.05 for the two replicates, respectively. A summary of results from cross validation for the EBLASSO-NE, the EBLASSO-NEG and LASSO is given in Table S3. The estimated sizes of true effects and their standard errors are depicted along with all 20 true effects in Tables S1 and S2 for the two replicates, respectively.

The HyperLasso method: Hyperparameters for the HyperLasso and the corresponding number of effects identified for the two replicates are presented in Table S4. The best results in Table S4 include 10 true and 0 false positive effects for replicates 2 and 3. The estimated sizes of true effects and their standard errors for the best results in Table S4 are depicted along with all 20 true effects in Tables S1and S2 for the two replicates.

The BhGLM method: The 15 different pairs of values for ν_i and τ_i as listed in Table S5 were examined for the BhGLM method. With $\nu_i = 10^{-3}$, 10^{-4} and 10^{-5}, $\tau_i = 10^{-4}$, the best results include 11 true and 0 false positive effects for replicates 2, and 9 true and 1 false positive effects for replicate 3. The corresponding estimated sizes of the true effects and their standard errors are depicted in Tables S1 and S2 for the two replicates, respectively.

The RVM method: The MATLAB implementation of the RVM identified 30 and 32 nonzero effects with a *p*-value ≤ 0.05 for the two replicates, respectively. Among these effects, we obtained 15 true and 15 false positive for replicate 1, 16 true and 16 false positive for replicate 2. The estimated sizes of true effects and their standard errors are depicted in Tables S1and S2. When we reduced the number of false positive effects to three, at a level slightly higher than that of our EBLASSO-NEG and EBLASSO-NE, by reducing the cutoff for the *p*-

value, we obtained 11 and 10 positive effects for the two replicates, which are smaller than the number of true effects identified with our EBLASSO-NEG and EBLASSO-NE.

The single QTL method: After Bonferroni correction, effects with a *p*-value $\leq$ 0.05/481 = 1.04×10^{-4} were considered as significant, which identified 7 true and 22 false positive effects for replicate 2, and 7 true and 13 false positive effects for replicate 3. The estimated sizes of true effects and their standard errors are depicted in Tables S1and S2, respectively, for the two replicates. When we increased the number of true positive effects to 13, at a level same as our EBLASSO-NEG or EBLASSO-NE, by increasing the cutoff for the *p*-value to 3.10×10^{-3} for replicate 2 and 2.68×10^{-2} for replicate 3, we obtained 27 and 41 false positive effects, respectively.

As shown in Tables S1 and S2, the EBLASSO-NE and the EBLASSO-NEG identified more true effects than four other methods except the RVM, and yielded a number of false positive effects comparable to those of the LASSO, the HyperLasso and the BhGLM but much smaller than those of the RVM and the single-QTL mapping method.

All simulations were performed on a personal computer (PC) with a 3.4 GHz Intel PentiumD CPU and 2Gb memory running Windows XP, except that the HyperLasso was ran on an IBM BladeCenter cluster including computing nodes with 2.6 GHz Xeon or 2.2 GHz Opteron CPU running Linux. The speeds of the EBLASSO-NEG, the LASSO and the HyperLasso were comparable and faster than the other methods. The speeds of the EBLASSO-NE, the BhGLM and the single-QTL mapping method were comparable but are much faster than the RVM.

Replicates 2 and 3 for the simulations with main and epistatic effects

In this simulation setup, 10 main and 10 epistatic effects were simulated. The genotypes of m = 481 markers of n = 1,000 individuals were generated independently using the procedure

described in the main text. Locations and effects of the QTLs and QTL pairs are shown in Table S6. The QTL model contained a total of $k = 1 + 481 + \binom{481}{2} = 115{,}922$ possible effects, a number about 116 times of the sample size, and the design matrix **X** was of size $1000 \times 115{,}921$. QTL mapping was performed with all methods described in the previous simulations. However, like the simulations for replicate 1, the BhGLM method did not converge and the RVM ran out of memory due to a large number of nonzero effects included in the model, and thus, they did not yield any result.

The same cross-validation procedures described earlier were performed to choose the optimal values of the hyperparameters for the EBLASSO-NE, the EBLASSO-NEG and LASSO, and the results for several values of hyperparameters are presented in Table S8. Optimal values of the hyperparameters were then used to analyze the whole dataset. The number of true and false positive effects identified and the estimated effect sizes of the detected true effects are given in Tables S6 and S7, respectively, for the two replicates.

For the HyperLasso, we again examined 12 pairs of values for hyperparameters *a* and *b* as listed in Table S9, and identified $a = 0.1$ and $b = 4.9 \times 10^{-4}$ as the optimal values that gave best tradeoff between the true and false positive effects. For the two-locus test, effects with a *p*-value $\leq 0.05/k = 4.31 \times 10^{-7}$ were considered as significant. Detailed QTL mapping results for the HyperLasso and the single-QTL mapping method are also given in Tables S6 and S7, respectively, for the two replicates.

As shown in Tables S6 and S7, the EBLASSO-NE and the EBLASSO-NEG detected the same or a larger number of true effects but a smaller number of false positive than the other methods, which demonstrates that our EBLASSO-NE and EBLASSO-NEG offer the best overall

performance in terms of power of detection and the false positive rate. The LASSO is the fastest, and the EBLASSO-NEG and the HyperLasso are the second and the third fastest.

Table S1 True and estimated effects for the simulated data with main effects (Replicate 2).

locus	True β	EBLASSO-NE $\hat{\beta}(s_{\hat{\beta}})^a$	EBLASSO-NEG $\hat{\beta}(s_{\hat{\beta}})^a$	LASSO $\hat{\beta}(s_{\hat{\beta}})^a$	HyperLasso $\hat{\beta}(s_{\hat{\beta}})^a$	BhGLM $\hat{\beta}(s_{\hat{\beta}})^a$	RVM $\hat{\beta}(s_{\hat{\beta}})^a$	Single QTL $\hat{\beta}(s_{\hat{\beta}})^a$
11	1.99	0.87(0.30)	2.07(0.29)	–	1.32(0.62)	1.93(0.29)	3.53(0.78)	0.84(0.14)
26	1.81	1.44(0.22)	1.95(0.29)	1.10(0.59)	2.24(0.32)	2.12(0.30)	4.06(0.62)	0.90(0.14)
42	-1.28	-0.41(0.24)[b]	-0.54(0.27)	–	-1.08(0.45)	1.43(0.30)[b]	-1.84(0.63)[b]	–
48	-0.91	-0.41(0.20)	-0.60(0.27)	-0.27(0.58)	-1.00(0.44)[b]	0.73(0.27)	–	–
72	1.28	0.75(0.34)	1.74(0.51)	–	1.49(0.42)[b]	-1.36(0.39)[b]	–	1.04(0.14)
73	1.81	1.21(0.32)	1.75(0.49)	0.84(1.04)	2.17(0.45)	-1.99(0.41)	4.13(0.70)	0.96(0.13)
123	0.63	–	–	–	–	–	1.13(0.38)	–
127	-0.63	–	–	–	–	–	–	–
161	0.44	–	–	–	–	–	–	–
181	0.99	–	–	–	–	–	–	1.65(0.17)
182	2.19	2.57(0.39)	4.26(0.50)	1.90(1.23)	4.93(0.53)	-3.88(0.51)	6.44(1.00)	1.91(0.19)
185	1.29	0.50(0.22)	–	0.42(0.84)	–	-0.80(0.35)	–	1.58(0.17)
221	-0.75	-0.31(0.16)	-0.34(0.19)	-0.22(0.42)	-0.65(0.25)	-0.57(0.21)	-1.18(0.38)	–
243	-0.57	–	–	–	–	–	–	–
262	-1.28	-0.47(0.19)	-0.79(0.26)	-0.23(0.39)	-0.89(0.25)	-0.76(0.23)	-2.08(0.47)	–
268	0.91	0.30(0.18)[b]	0.75(0.28)[b]	–	–	–	2.22(0.50)[b]	–
270	0.57	–	–	–	–	–	–	–
274	-0.99	–	-0.57(0.25)	-0.01(0.45)	–	–	-2.07(0.48)	–
361	0.41	0.38(0.19)[b]	0.95(0.23)[b]	–	0.90(0.24)[b]	-0.72(0.22)[b]	–	–
461	0.51	–	0.40(0.19)[b]	0.03(0.56)[b]	–	–	1.50(0.38)	–
Parameter(s)		$\lambda = 0.0224$	a = -0.5<br>b = 10	$\lambda = 0.0197$	$a = 0.01$<br>$\alpha = 0.05$	$\upsilon = 10^{-3}$<br>$s = 10^{-4}$		
CPU time(s)		19.78	4.25	2.02	2.10	19.81	39.71	8.46
True/False positive		12/0[c]	13/3[c]	9/4[c]	10/0[c]	11/0[c]	15/15[c]	7/22[d]

[a]The estimated marker effect is denoted by $\hat{\beta}$ and the standard deviation is denoted by $s_{\hat{\beta}}$.

[b]The estimated marker effect was obtained from a neighboring marker (≤ 20 cM) rather than from the marker with true effect.

[c]Number of effects with a *p*-value ≤ 0.05.

[d]Number of effects with a p-value ≤ 1.04×10^{-4} after Bonferroni correction was applied.

Table S2 True and estimated effects for the simulated data with main effects (Replicate 3).

locus	True β	EBLASSO-NE $\hat{\beta}(s_{\hat{\beta}})$[a]	EBLASSO-NEG $\hat{\beta}(s_{\hat{\beta}})$[a]	LASSO $\hat{\beta}(s_{\hat{\beta}})$[a]	HyperLasso $\hat{\beta}(s_{\hat{\beta}})$[a]	BhGLM $\hat{\beta}(s_{\hat{\beta}})$[a]	RVM $\hat{\beta}(s_{\hat{\beta}})$[a]	Single QTL $\hat{\beta}(s_{\hat{\beta}})$[a]
11	1.99	0.88(0.26)	1.49(0.24)	–	0.61(0.19)	1.48(0.24)	–	0.67(0.14)
26	1.81	1.12(0.19)	1.46(0.23)	0.75(0.35)	0.86(0.20)	1.48(0.24)	2.75(0.41)	–
42	-1.28	-0.74(0.21)	-1.02(0.24)	-0.52(0.39)	-1.08(0.24)	1.09(0.25)	-2.70(0.69)	–
48	-0.91	-0.53(0.24)[b]	-1.17(0.24)[b]	–	-0.74(0.24)	-1.07(0.25)	-3.15(0.47)[b]	-0.52(0.13)
72	1.28	1.10(0.33)	2.47(0.28)	0.82(0.84)	1.27(0.41)	1.79(0.44)	4.09(0.53)	1.08(0.15)
73	1.81	0.60(0.28)	–	–	–	–	–	1.10(0.15)
123	0.63	–	–	–	–	–	–	–
127	-0.63	–	–	–	–	–	–	–
161	0.44	–	–	0.08(0.31)[b]	–	–	–	–
181	0.99	0.41(0.24)	–	0.28(0.63)	–	–	–	1.51(0.16)
182	2.19	1.72(0.34)	2.77(0.37)	1.41(0.75)	1.91(0.28)	-2.64(0.38)	4.67(0.90)	1.70(0.17)
185	1.29	0.78(0.24)	0.76(0.28)	0.59(0.44)	0.62(0.26)	1.03(0.30)	–	1.29(0.15)
221	-0.75	-0.71(0.17)	-0.87(0.20)	-0.50(0.29)	-0.50(0.19)	-1.08(0.21)	-3.28(0.57)	–
243	-0.57	–	-0.49(0.18)[b]	–	-0.56(0.19)	–	-1.51(0.33)[b]	–
262	-1.28	-0.54(0.19)	-0.77(0.22)	-0.28(0.34)	-0.67(0.19)	-0.83(0.21)	-2.04(0.49)	–
268	0.91	0.41(0.20)[b]	0.72(0.25)[b]	0.01(0.43)[b]	–	–	2.52(0.54)[b]	–
270	0.57	–	–	–	–	–	–	–
274	-0.99	-0.59(0.20)	-0.86(0.24)	-0.19(0.39)	–	–	-2.70(0.49)	–
361	0.41	–	–	–	–	–	–	–
461	0.51	–	–	–	–	–	–	–
Parameter(s)		$\lambda = 0.0224$	a = -0.4<br>b = 6	$\lambda = 0.0213$	$a = 0.1$<br>$\alpha = 0.05$	$\upsilon = 10^{-3}$<br>$s = 10^{-4}$		
CPU time(s)		40.41	3.79	1.50	1.10	12.22	48.46	34.02
True/False positive		13/1[c]	12/2[c]	11/2[c]	10/0[c]	9/1[c]	16/16[c]	7/13[d]

[a]The estimated marker effect is denoted by $\hat{\beta}$ and the standard deviation is denoted by $s_{\hat{\beta}}$.

[b]The estimated marker effect was obtained from a neighboring marker (≤ 20 cM) rather than from the marker with true effect.

[c]Number of effects with a *p*-value ≤ 0.05.

[d]Number of effects with a p-value ≤ 1.04×10^{-4} after Bonferroni correction was applied.

Table S3 Cross-validations of the EBLASSO-NE, EBLASSO-NEG and LASSO for the simulations with only main effects.

Algorithm	Replicate 2		Replicate 3	
	Parameters[a]	logL ± STE[b]	Parameters[a]	logL ± STE[b]
EBLASSO-NE	0.0028	-0.32 ± 0.04	0.0028	-0.42 ± 0.03
	0.0056	-0.34 ± 0.04	0.0056	-0.43 ± 0.04
	0.0112	-0.35 ± 0.05	0.0112	-0.42 ± 0.02
	0.0224	-0.29 ± 0.03[c]	0.0224	-0.34 ± 0.02[c]
	0.0447	-0.40 ± 0.05	0.0447	-0.36 ± 0.03
	0.0891	-0.36 ± 0.02	0.0891	-0.39 ± 0.03
	0.1778	-0.38 ± 0.04	0.1778	-0.39 ± 0.02
EBLASSO-NEG	(-0.5,0.01)	-0.31 ± 0.03	(-0.5,0.1)	-0.42 ± 0.05
	(0.5,0.01)	-0.36 ± 0.03	(-0.4,0.1)	-0.38 ± 0.02
	(1,0.01)	-0.38 ± 0.02	(-0.3,0.1)	-0.43 ± 0.03
	(-0.5,4)	-0.32 ± 0.05	(-0.4,5)	-0.42 ± 0.05
	(-0.5,8)	-0.32 ± 0.05	(-0.4,6)	--0.33 ± 0.02[c]
	(-0.5,10)	-0.31 ± 0.05[c]	(-0.4,7)	-0.36 ± 0.03
LASSO	0.0796	-0.45 ± 0.02	0.0861	-0.53 ± 0.02
	0.0396	-0.36 ± 0.03	0.0460	-0.42 ± 0.03
	0.0197	-0.30 ± 0.03	0.0213	-0.36 ± 0.04
	0.0113	-0.29 ± 0.04[c]	0.0131	-0.34 ± 0.04[c]
	0.0065	-0.30 ± 0.05	0.0070	-0.35 ± 0.06

[a]Parameters are λ for EBLASSO-NE and LASSO, (a, b) for EBLASSO-NEG.

[b]The average log likelihood and standard error were obtained from ten-fold cross validation.

[c]The optimal log likelihood and corresponding parameter(s) chosen for comparison with other methods.

Table S4 Summary of results of the HyperLasso for the simulations with only main effects.

Parameters			True/False positive effects[a]	
Shape *a*	Inverse scale *b*	Type I error α	Replicate 2	Replicate 3
0.1	1.7×10^{-3}	0.05	9/0	10/0[b]
0.05	1.5×10^{-3}		10/0	10/0
0.01	1.4×10^{-3}		10/0[b]	10/0
0.1	9.8×10^{-4}	0.01	6/0	9/0
0.05	8.8×10^{-4}		6/0	9/0
0.01	7.9×10^{-4}		6/0	9/0
0.1	5.2×10^{-4}	$\frac{0.05}{481}$	5/0	6/0
0.05	4.7×10^{-4}		6/0	6/0
0.01	4.2×10^{-4}		6/0	7/0
0.1	3.6×10^{-4}	$\frac{0.01}{481}$	4/0	4/0
0.05	3.2×10^{-4}		4/0	4/0
0.01	2.9×10^{-4}		4/0	4/0

[a]Effects with *p*-value ≤ 0.05 were considered as significant different from zero.

[b]The optimal results chosen for comparison with other methods.

Table S5 Summary of results of the BhGLM for the simulations with only main effects.

Parameters		True/False positive effects[a]	
ν	τ	Replicate 2	Replicate 3
10^{-5}		11/0	9/1
10^{-4}		11/0	9/1
10^{-3}	10^{-5}	11/0	9/1
10^{-2}		10/0	9/1
10^{-1}		8/0	9/1
10^{-5}		11/0	9/1
10^{-4}		11/0	9/1
10^{-3}	10^{-4}	11/0[b]	9/1[b]
10^{-2}		10/0	9/1
10^{-1}		8/0	9/1
10^{-5}		11/0	9/1
10^{-4}		11/0	9/1
10^{-3}	10^{-3}	11/0	9/1
10^{-2}		10/0	9/1
10^{-1}		8/0	9/1

[a]Effects with *p*-value ≤ 0.05 were considered as significant different from zero.

[b]The optimal results chosen for comparison with other methods.

Table S6 True and estimated effects for the simulated data with main and epistatic effects (Replicate 2).

locus *i*	locus *j*	True β	EBLASSO-NE $\hat{\beta}(s_{\hat{\beta}})^a$	EBLASSO-NEG $\hat{\beta}(s_{\hat{\beta}})^a$	LASSO $\hat{\beta}(s_{\hat{\beta}})^a$	HyperLasso $\hat{\beta}(s_{\hat{\beta}})^a$	Two-locus test $\hat{\beta}(s_{\hat{\beta}})^a$
11	11	1.99	0.94(0.13)	1.64(0.18)	0.67(0.42)	2.21(0.24)	0.73(0.10)
26	26	1.81	0.99(0.14)	1.77(0.18)	0.69(0.65)	2.29(0.25)	0.72(0.10)
42	42	-1.28	-0.41(0.13)	-0.88(0.19)	-0.29(0.47)	-1.24(0.25)	−
48	48	-0.91	-0.54(0.13)[b]	-0.81(0.19)[b]	-0.27(0.82)[b]	-1.02(0.26)[b]	−
72	72	1.28	1.18(0.19)	2.65(0.20)	0.84(0.70)	2.09(0.45)	1.02(0.10)
73	73	1.81	0.63(0.18)	−	0.46(0.72)	1.56(0.44)	0.99(0.10)
182	182	2.19	1.41(0.18)	2.13(0.25)	1.10(0.64)	2.76(0.35)	1.35(0.11)
185	185	1.29	0.74(0.16)	1.03(0.22)	0.47(0.59)	1.51(0.31)	1.21(0.11)
262	262	-1.28	-0.18(0.10)	-0.53(0.14)	-0.07(0.35)	−	−
268	268	0.91	−	−	−	−	−
5	6	1.28	0.19(0.11)	0.84(0.20)	−	−	−
6	39	1.29	−	−	−	−	−
42	220	1.99	0.72(0.18)	1.42(0.23)	0.02(1.47)	−	−
81	200	-1.28	-0.37(0.15)[b]	-0.70(0.20)	−	-1.09(0.28)[b]	−
87	164	1.81	0.48(0.17)	1.15(0.22)	0.25(1.04)	1.65(0.28)[b]	−
87	322	2.19	0.70(0.20)	1.98(0.23)	0.16(1.22)[b]	2.78(0.32)	0.76(0.13)
118	278	-1.28	-0.44(0.15)	-1.06(0.20)	-0.22(0.47)	-1.10(0.26)	−
328	404	-0.99	-0.37(0.14)[b]	-0.94(0.20)[b]	-0.09(0.56)[b]	-1.18(0.26)[b]	−
373	400	-0.91	-0.27(0.14)	-0.86(0.20)	-0.12(1.30)	-1.08(0.28)[b]	−
431	439	1.81	0.39(0.15)[b]	0.92(0.20)[b]	−	1.23(0.28)[b]	−
Parameter(s)			$\lambda = 0.0891$	$a = -0.01$, $b = 1$	$\lambda = 0.0273$	$a = 0.1$, $\alpha = 0.01$	
CPU time(s)			3285.8	293.3	80.6	626.2	2922.3
True/False positive			18/9[c]	17/4[c]	15/32[c]	15/11[c]	7/15[d]

[a]The estimated marker effect is denoted by $\hat{\beta}$ and the standard deviation is denoted by $s_{\hat{\beta}}$.

[b]The estimated marker effect was obtained from a neighboring marker ($\leq$ 20 cM) rather than from the marker with true effect.

[c]Number of effects with p-value ≤ 0.05.

[d]Number of effects with a p-value $\leq 4.31\times10^{-7}$ after Bonferroni correction was applied.

Table S7 True and estimated effects for the simulated data with main and epistatic effects (Replicate 3).

locus *i*	locus *j*	True β	EBLASSO - NE $\hat{\beta}(s_{\hat{\beta}})^a$	EBLASSO - NEG $\hat{\beta}(s_{\hat{\beta}})^a$	LASSO $\hat{\beta}(s_{\hat{\beta}})^a$	HyperLasso $\hat{\beta}(s_{\hat{\beta}})^a$	Two-locus test $\hat{\beta}(s_{\hat{\beta}})^a$
11	11	1.99	0.81(0.12)	1.67(0.17)	0.68(0.82)	1.62(0.27)	0.89(0.10)
26	26	1.81	0.75(0.13)	1.74(0.18)	0.68(1.07)	1.86(0.21)	0.77(0.10)
42	42	-1.28	-0.51(0.12)[b]	-0.99(0.19)[b]	–	-1.02(0.22)[b]	–
48	48	-0.91	-0.34(0.11)	-0.88(0.19)	–	-1.09(0.23)	–
72	72	1.28	0.48(0.14)	–	0.46(1.1)	1.12(0.43)	0.98(0.10)
73	73	1.81	0.78(0.16)	2.65(0.21)	0.61(1.32)	1.39(0.41)	1.00(0.10)
182	182	2.19	0.88(0.15)	2.05(0.25)	0.79(1.11)	2.41(0.30)	1.09(0.10)
185	185	1.29	0.67(0.14)	1.22(0.23)	0.63(0.94)	1.52(0.27)	1.14(0.11)
262	262	-1.28	-0.21(0.09)	-0.54(0.14)[b]	–	–	–
268	268	0.91	–	–	–	–	–
5	6	1.28	0.27(0.11)	1.06(0.21)	0.32(0.67)	1.19(0.25)[b]	–
6	39	1.29	0.35(0.13)[b]	0.98(0.21)	0.24(0.71)[b]	1.30(0.24)[b]	–
42	220	1.99	0.44(0.16)	1.28(0.23)	0.11(0.84)[b]	1.62(0.27)[b]	–
81	200	-1.28	–	-0.83(0.20)	–	–	–
87	164	1.81	0.37(0.14)	1.15(0.23)	0.31(1.06)[b]	0.70(0.35)[b]	–
87	322	2.19	0.74(0.16)	1.96(0.24)	0.60(0.83)	2.23(0.28)	0.82(0.14)
118	278	-1.28	-0.28(0.12)[b]	-1.00(0.22)[b]	–	-1.30(0.26)[b]	–
328	404	-0.99	-0.18(0.11)	–	–	-0.91(0.24)	–
373	400	-0.91	–	–	–	–	–
431	439	1.81	0.57(0.15)	1.67(0.22)	–	1.53(0.29)[b]	0.64(0.13)
Parameter(s)			$\lambda = 0.1778$	*a* = -0.3<br>*b* = 0.05	$\lambda = 0.0254$	a=0.1<br>α=0.01	
CPU time(s)			1068.1	351.0	103.5	682.7	2949.3
True/False positive			17/9[c]	16/4[c]	11/16[c]	16/6[c]	8/16[d]

[a]The estimated marker effect is denoted by $\hat{\beta}$ and the standard deviation is denoted by $s_{\hat{\beta}}$.

[b]The estimated marker effect was obtained from a neighboring marker (≤ 20 cM) rather than from the marker with true effect.

[c]Number of effects with *p*-value ≤ 0.05.

[d]Number of effects with a *p*-value ≤ 4.31×10^{-7} after Bonferroni correction was applied.

Table S8 Cross-validations of the EBLASSO-NE, EBLASSO-NEG and LASSO for the simulations with main and epistatic effects.

**Algorithm**	**Replicate 2**		**Replicate 3**	
	Parameters[a]	logL ± STE[b]	Parameters[a]	logL ± STE[b]
EBLASSO-NE	0.0316	-0.41 ± 0.03	0.0631	-0.40 ± 0.03
	0.0447	-0.45 ± 0.03	0.0891	-0.37 ± 0.01
	0.0631	-0.42 ± 0.04	0.1259	-0.42 ± 0.04
	0.0891	-0.40 ± 0.04[c]	0.1778	-0.38 ± 0.01[c]
	0.1259	-0.46 ± 0.05	0.2512	-0.43 ± 0.04
	0.1778	-0.48 ± 0.04	0.3548	-0.42 ± 0.03
	0.2512	-0.44 ± 0.03	0.5012	-0.42 ± 0.01
EBLASSO-NEG	(-0.1,0.01)	-0.38 ± 0.02	(-0.5,0.05)	-0.43 ± 0.03
	(-0.01,0.01)	-0.37 ± 0.01	(-0.2,0.05)	-0.38 ± 0.02
	(0.1,0.01)	-0.42 ± 0.03	(-0.1,0.05)	-0.43 ± 0.03
	(-0.01,0.5)	-0.47 ± 0.04	(-0.3,0.01)	-0.39 ± 0.02
	(-0.01,1)	-0.36 ± 0.02[c]	(-0.3,0.05)	-0.37 ± 0.02[c]
	(-0.01,2)	-0.41 ± 0.04	(-0.3,0.1)	-0.38 ± 0.03
LASSO	0.1100	-0.59 ± 0.01	0.1026	-0.59 ± 0.01
	0.0548	-0.47 ± 0.02	0.0511	-0.47 ± 0.02
	0.0273	-0.39 ± 0.02[c]	0.0254	-0.39 ± 0.02[c]
	0.0136	-0.39 ± 0.03	0.0126	-0.38 ± 0.03
	0.0068	-0.40 ± 0.04	0.0063	-0.39 ± 0.04

[a]Parameters are λ for EBLASSO-NE and LASSO, (a, b) for EBLASSO-NEG.

[b]The average log likelihood and standard error were obtained from ten-fold cross validation.

[c]The optimal log likelihood and corresponding parameter(s) chosen for comparison with other methods.

Table S9 Summary of results of the HyperLasso for the simulations with main and epistatic effects.

Parameters			True/False positive effects[a]	
Shape *a*	Inverse scale *b*	Type I error α	Replicate 2	Replicate 3
0.1	8.5×10^{-4}	0.05	18/18	17/19
0.05	7.6×10^{-4}		18/18	17/19
0.01	6.8×10^{-4}		17/17	17/20
0.1	4.9×10^{-4}	0.01	15/11[b]	16/6[b]
0.05	4.4×10^{-4}		15/11	16/6
0.01	3.9×10^{-4}		15/11	16/6
0.1	1.3×10^{-4}	$\frac{0.05}{115921}$	9/0	9/0
0.05	1.1×10^{-4}		9/0	9/0
0.01	1.0×10^{-4}		9/0	10/0
0.1	1.1×10^{-4}	$\frac{0.01}{115921}$	9/0	9/0
0.05	1.0×10^{-4}		9/0	9/0
0.01	0.9×10^{-4}		9/0	9/0

[a]Effects with *p*-value ≤ 0.05 were considered as significant different from zero.

[b]The optimal results chosen for comparison with other methods.
